# Supplementary material for: Combinatorial Optimization of Protein Systems in Synthetic Cells
Source: ACS Synth Biol. 2026 Aug 4;15(8):3285–99. doi: 10.1021/acssynbio.6c00166 (PMC13505333; doi:10.1021/acssynbio.6c00166)
Supplement: Supplementary file 1 [file sb6c00166_si_001.pdf]

Supporting Information for

Combinatorial optimization of protein systems in synthetic cells

Marijn van den Brink[1], Nico J. Claassens[2] and Christophe Danelon[1,3*]

[1] Department of Bionanoscience, Kavli Institute of Nanoscience, Delft University of Technology, 2629 HZ Delft, The Netherlands

[2] Laboratory of Microbiology, Wageningen University, Stippeneng 4, 6708 WE Wageningen, The Netherlands

[3] Toulouse Biotechnology Institute (TBI), Université de Toulouse, CNRS, INRAE, INSA, 31077 Toulouse, France

* To whom correspondence should be addressed. Email: danelon@insa-toulouse.fr.

Content:

Pages S2-17 | Supplementary figures S1-20
Pages S18-26 | Supplementary tables S1-13

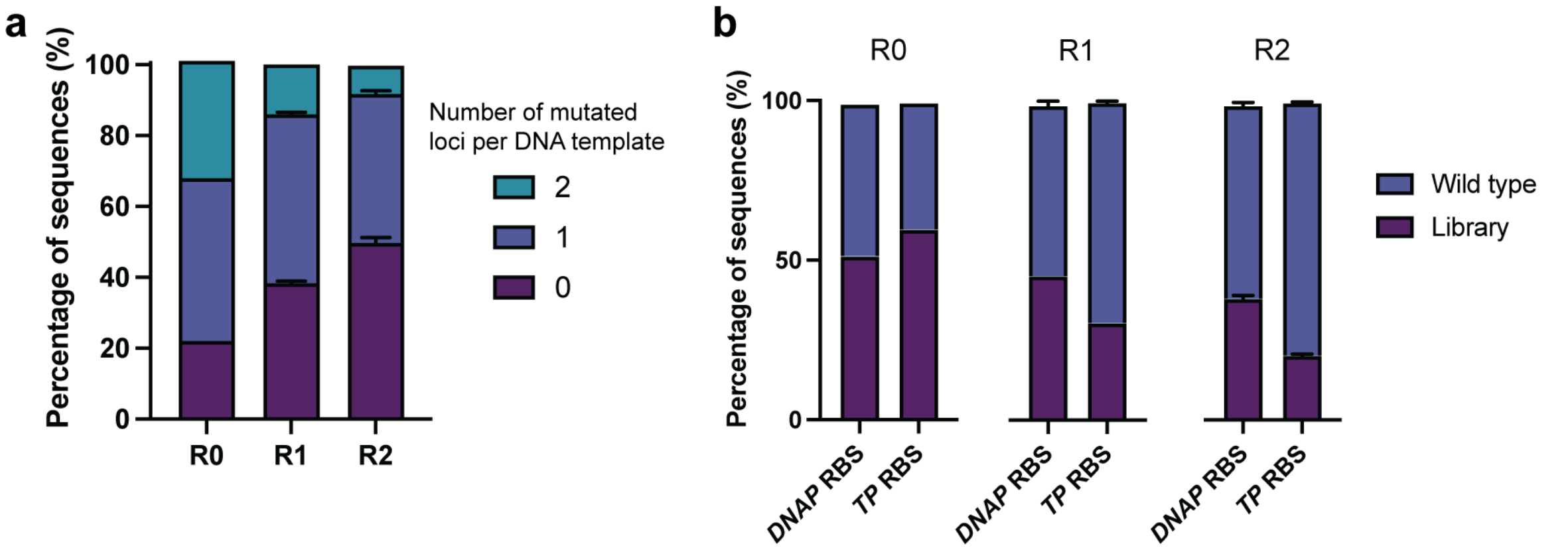

Fig. S1 Characterization of the orip2p3 RBS library before (R0) and after one (R1) or two (R2) rounds of IVTTR in liposomes. a Percentage of sequences with zero, one or two modified target loci. Percentage relative to the complete sequenced library after excluding reads with low-quality bases in the target sites. Number of biological replicates: n=1 (R0); n=3 (R1-2). Number of reads: 89,066 (R0); 72,884 (R1, replicate 1); 122,112 (R1, replicate 2); 72,501 (R1, replicate 3); 57,041 (R2, replicate 1); 76,140 (R2, replicate 2); 47,249 (R2, replicate 3). **b** Percentage of wild-type and modified sequences per target locus. The sums of percentages per bar for wild-type and intended library sequences add up to > 98%.

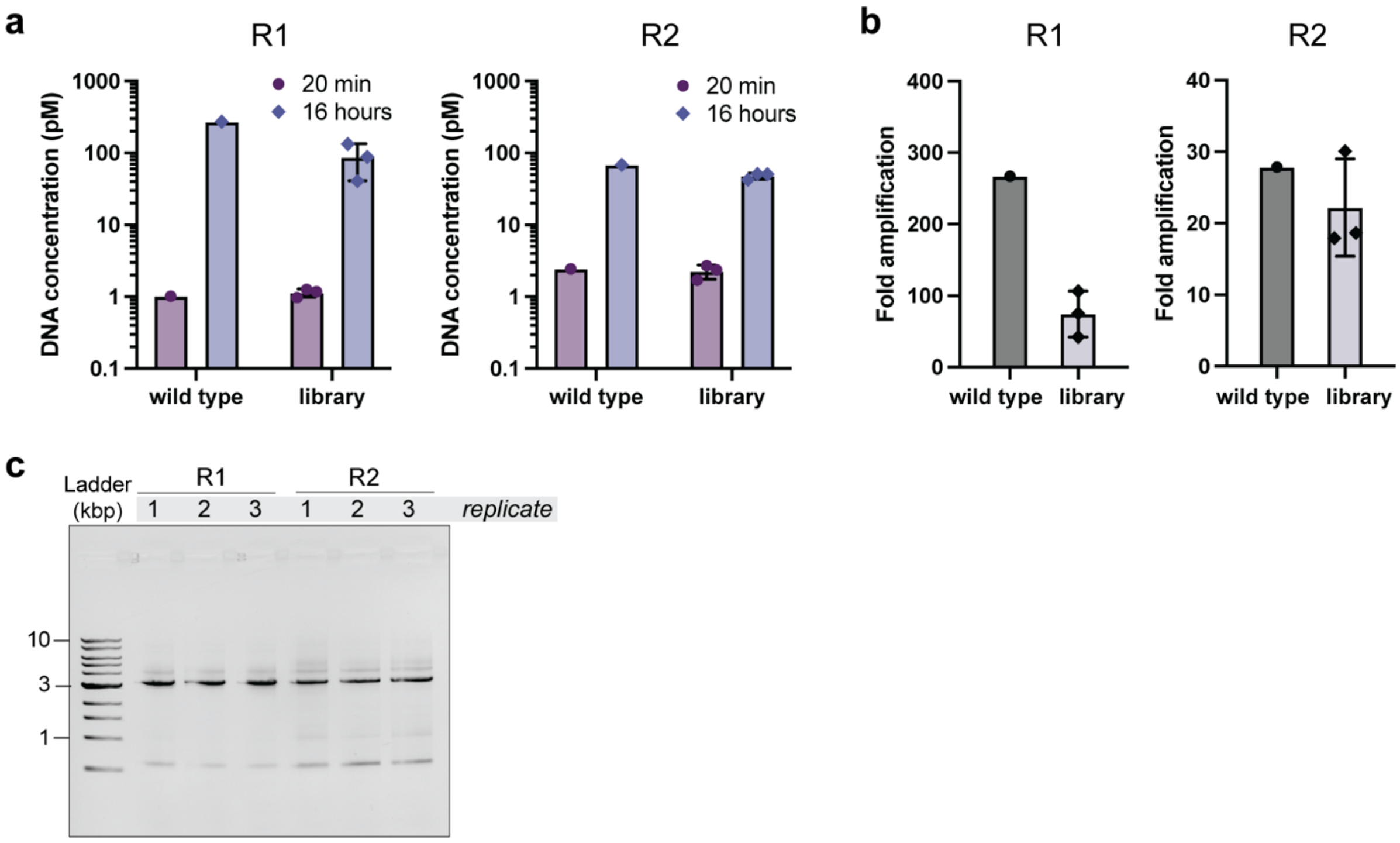

Fig. S2 Quantification and recovery of wild-type and library orip2p3 templates replicated in liposomes. a Absolute DNA quantification from in-liposome IVTTR reactions incubated for 16 hours or 20 min (negative control). **b** Replication fold from IVTTR reactions displayed in panel a. Replication fold was determined by the ratio of DNA concentration for reactions incubated for 16 h and 20 min. The wild type sample in round 2 (R2) was the wild-type template recovered from round 1 (R1). **c** PCR-amplified DNA templates (expected size: 3.2 kb) for library IVTTR after 16 h visualized by agarose gel electrophoresis. Purified PCR products were sequenced by nanopore sequencing.

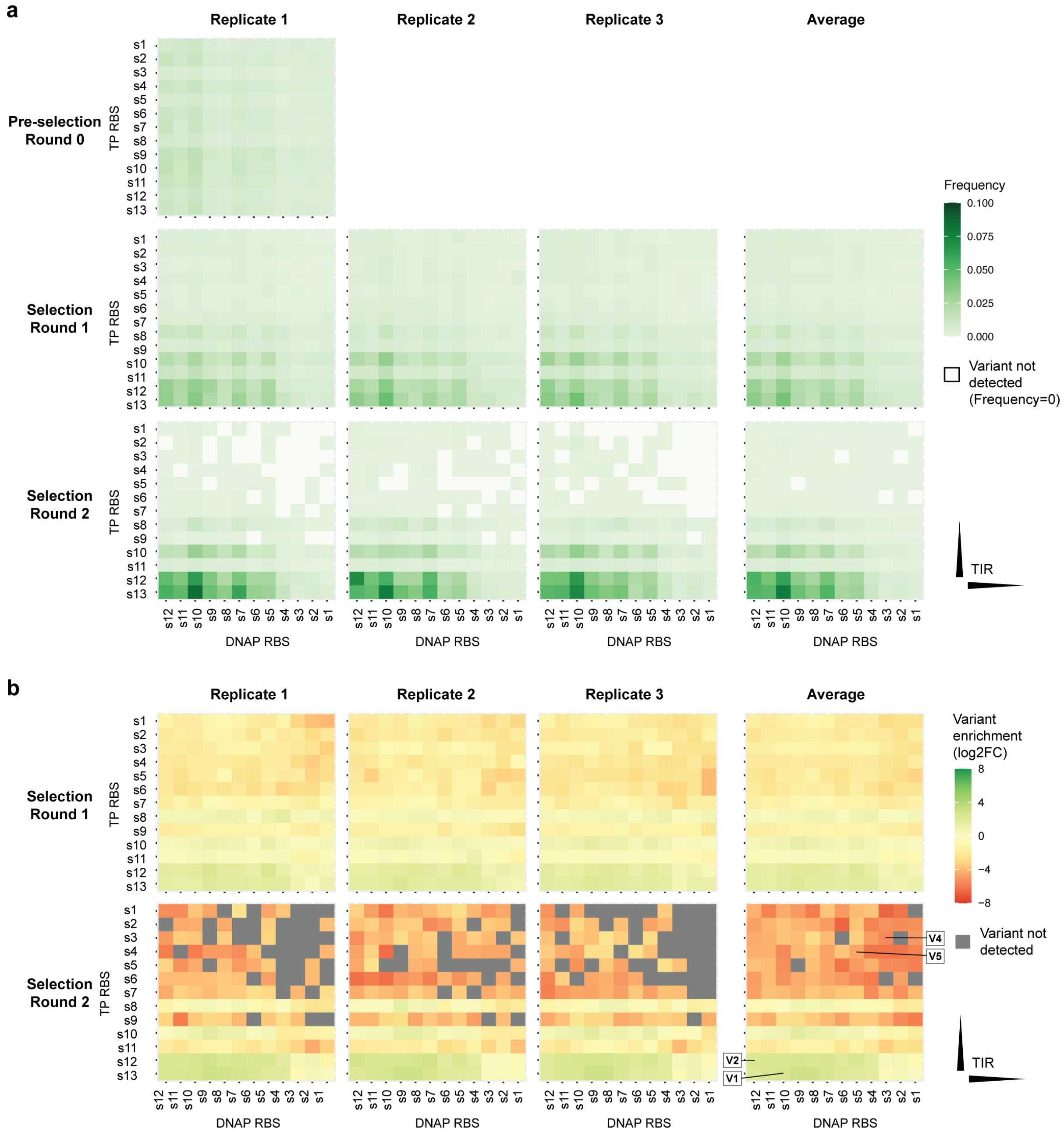

Fig. S3 Heatmaps displaying the frequency (a) or log2 fold change (b) of variants for each combination of *DNAP* and *TP* RBSs. a The frequency was calculated by dividing the number of reads by the total number of reads for the fraction of the library containing DNA variants with both RBSs mutated. Number of reads: 28,978 (R0); 10,436 (R1, replicate 1); 16,998 (R1, replicate 2); 10,140 (R1, replicate 3); 4,512 (R2, replicate 1); 6,060 (R2, replicate 2); 3,900 (R2, replicate 3). **b** To calculate the average log2FC, the average frequency was calculated, whereafter this value was divided by the initial frequency of the variant (round 0), and log2 transformed. Clonal DNA variants V1, V2, V4 and V5 are indicated in the right bottom heatmap. In panels a and b the variants are ranked from high to low predicted TIR.

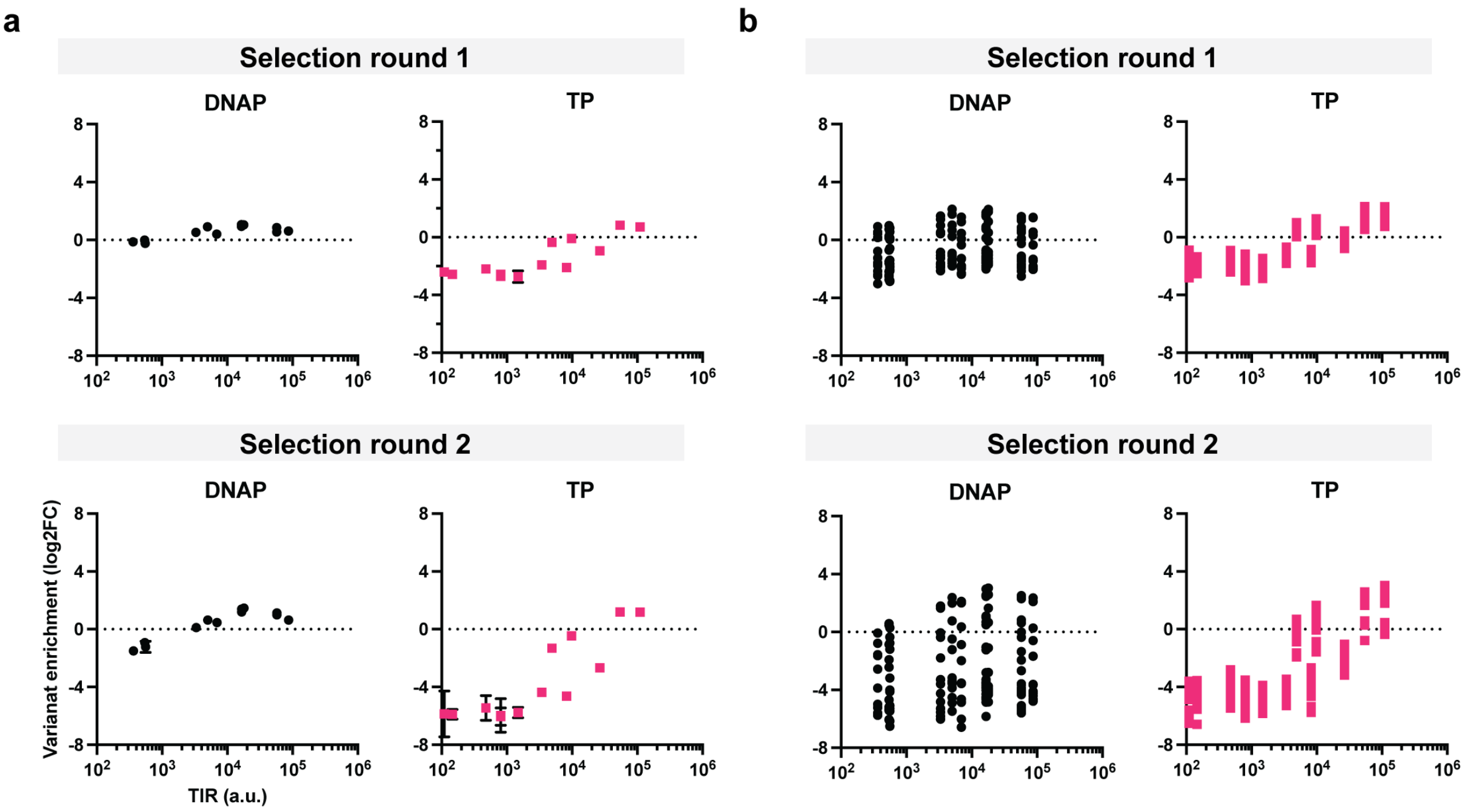

Fig. S4. Variant enrichment (log2FC) versus predicted TIR for rounds 1 and 2. a Individual data points for DNA variants that have one wild-type RBS and one mutated RBS. Error bars indicate standard deviation (n=3). **b** Data for the DNA variants that have two mutated RBSs (n=3). For each TIR value, there are multiple data points corresponding to the RBS variants of the other gene on the DNA template.

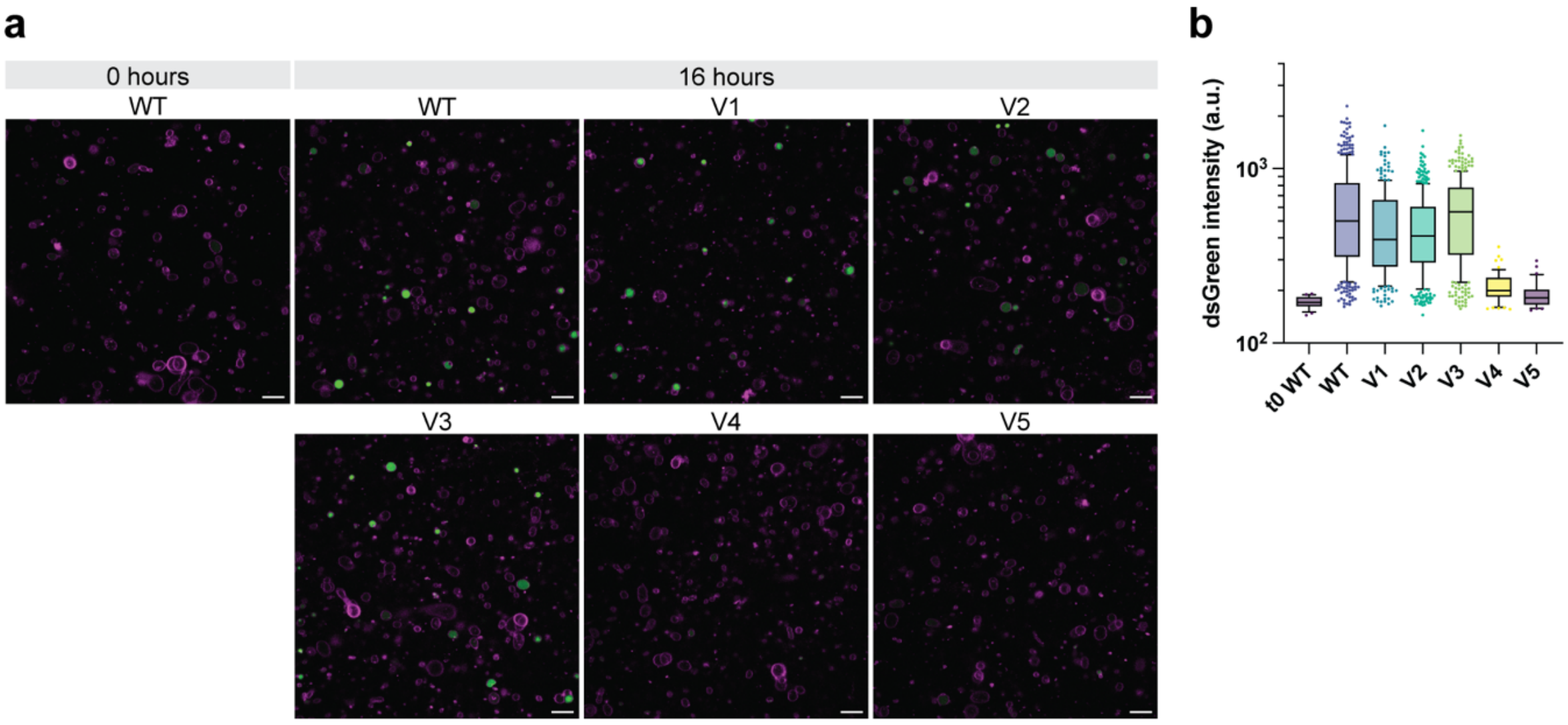

Fig. S5 Microscopy images of liposomes with replication of clonal DNA variants. a Additional microscopy images (partly identical to Fig. 1e). Magenta, Cy5-conjugated lipids; green, dsGreen; Scale bars: 10 µm. **b** Second biological replicate for dsGreen quantification in addition to replicate 1 shown in Fig. 1f. Number of dsGreen-positive liposomes: 23 (t0 WT), 364 (WT), 222 (V1), 356 (V2), 340 (V3), 55 (V4), 30 (V5).

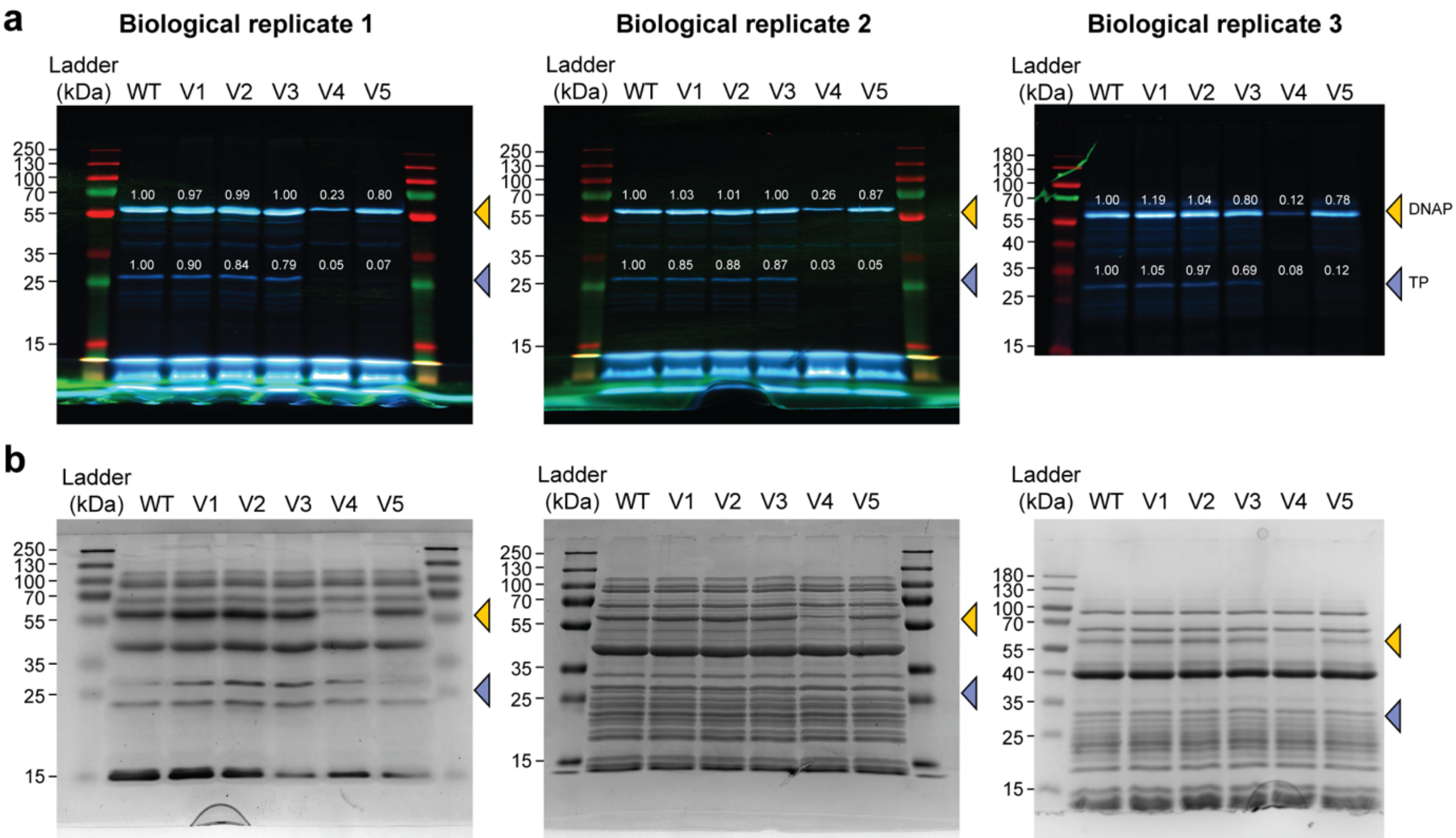

Fig. S6 Bulk PURE reactions with GreenLys protein labeling for orip2p3 DNA templates with different DNAP and TP RBSs. Biological replicate 1 was incubated for 8 hours at 30 °C. Biological replicates 2 and 3 were incubated for 2 hours at 30 °C. **a** Fluorescence imaging of SDS-PAGE gels for visualization of the bands corresponding to the proteins expressed by PURE system. Band intensities relative to the wild type (WT) are appended in the image. The gel for replicate 2 is also displayed in Fig. 1g. **b** Coomassie staining of the same gels shown in panel a to visualize the bands of all proteins. The staining of the gel for biological replicate 1 was most likely less sensitive due to the use of older Coomassie staining solution.

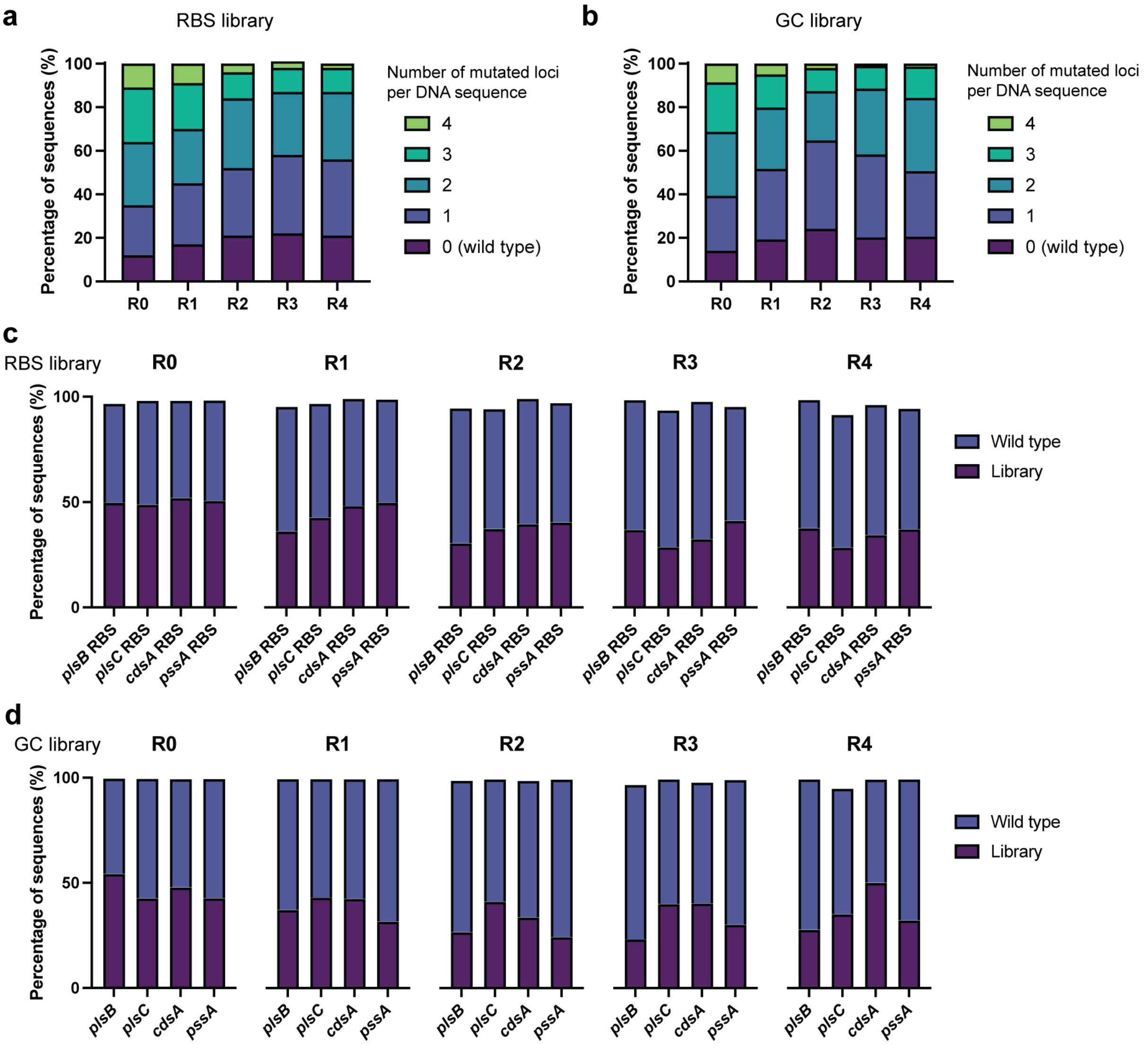

Fig. S7 Characterization of the phospholipid pathway RBS and GC libraries before (R0) and after one to four (R1-4) rounds of sorting of the liposomes with highest PS production by FACS. a-b Percentage of sequences with zero, one, two, three and four modified target loci for the RBS library (a) and GC library (b). Percentage relative to the complete sequenced library after excluding reads with low-quality bases in the target sites. Number of reads RBS library (a): 58,988 (R0); 77,727 (R1); 46,248 (R2); 31,776 (R3); 31,777 (R4). Number of reads GC library (b): 41,913 (R0); 116,996 (R1); 142,201 (R2); 118,839 (R3); 120,292 (R4). **c-d** Percentage of wild-type and modified sequences per target locus for the RBS library (c) and GC library (d).

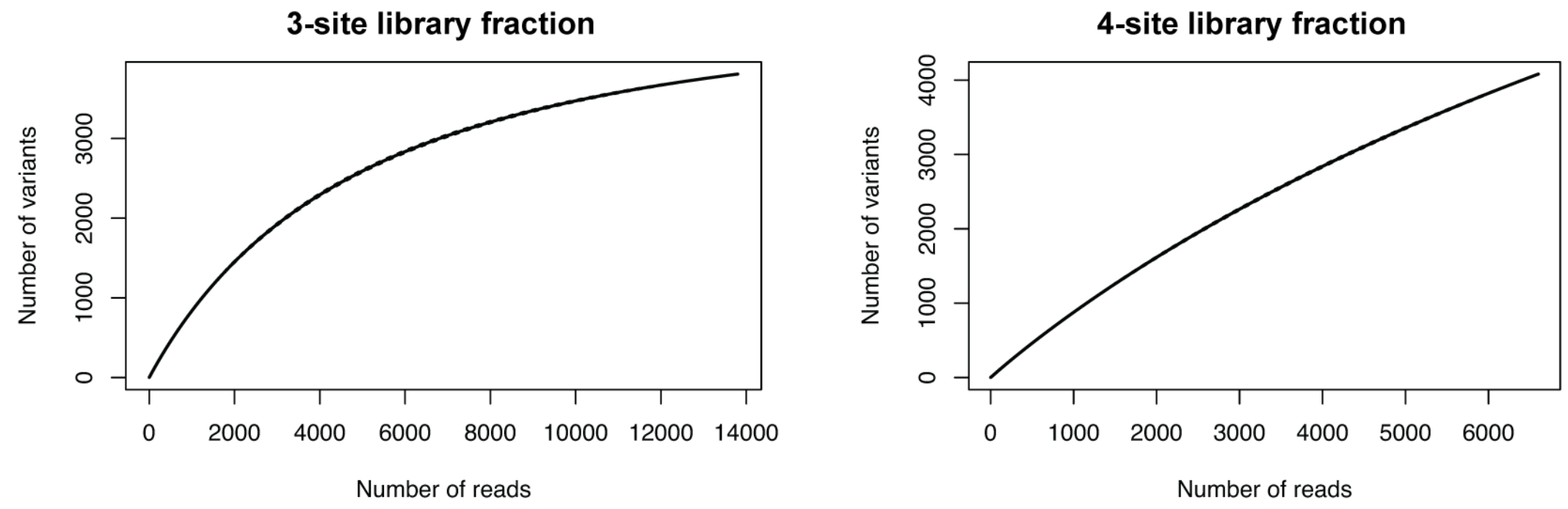

Fig. S8 Rarefaction plots for DNA variants with three or four RBSs mutated. Samples were an equimolar mix of four PCR-linearized plasmid library replicates. The graphs do not show a plateau, indicating that the library coverage is most likely larger than the number of variants detected in the nanopore sequencing dataset. For the DNA variants with three mutated RBSs, we detected 3,811 of the 4,518 variants (84%), based on 13,835 reads. For the DNA variants with four mutated RBSs, we detected 4,108 of the 11,583 variants (35%), based on 6,666 reads.

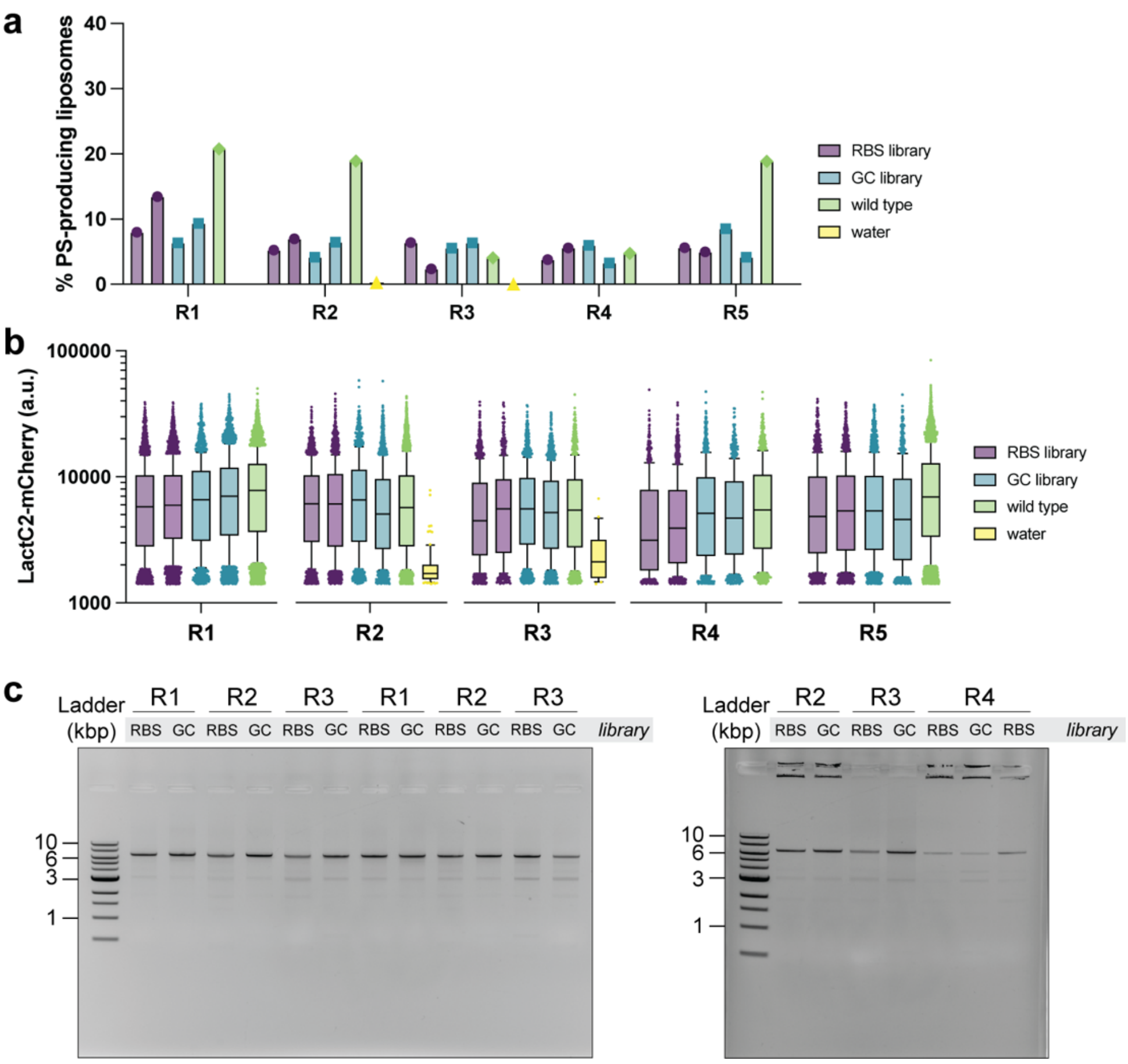

Fig. S9 FACS sorting and DNA recovery of liposomes producing PS. a Fraction of PS-producing liposomes measured by FACS. **b** LactC2-mCherry signal of the PS-producing liposomes measured by FACS. **a-b** For the RBS and GC libraries, two biological replicates were analyzed, indicated by identical bar colors. **c** Recovery of DNA from sorted liposomes by PCR (expected template length: 6.7 kb), visualized by agarose gel electrophoresis.

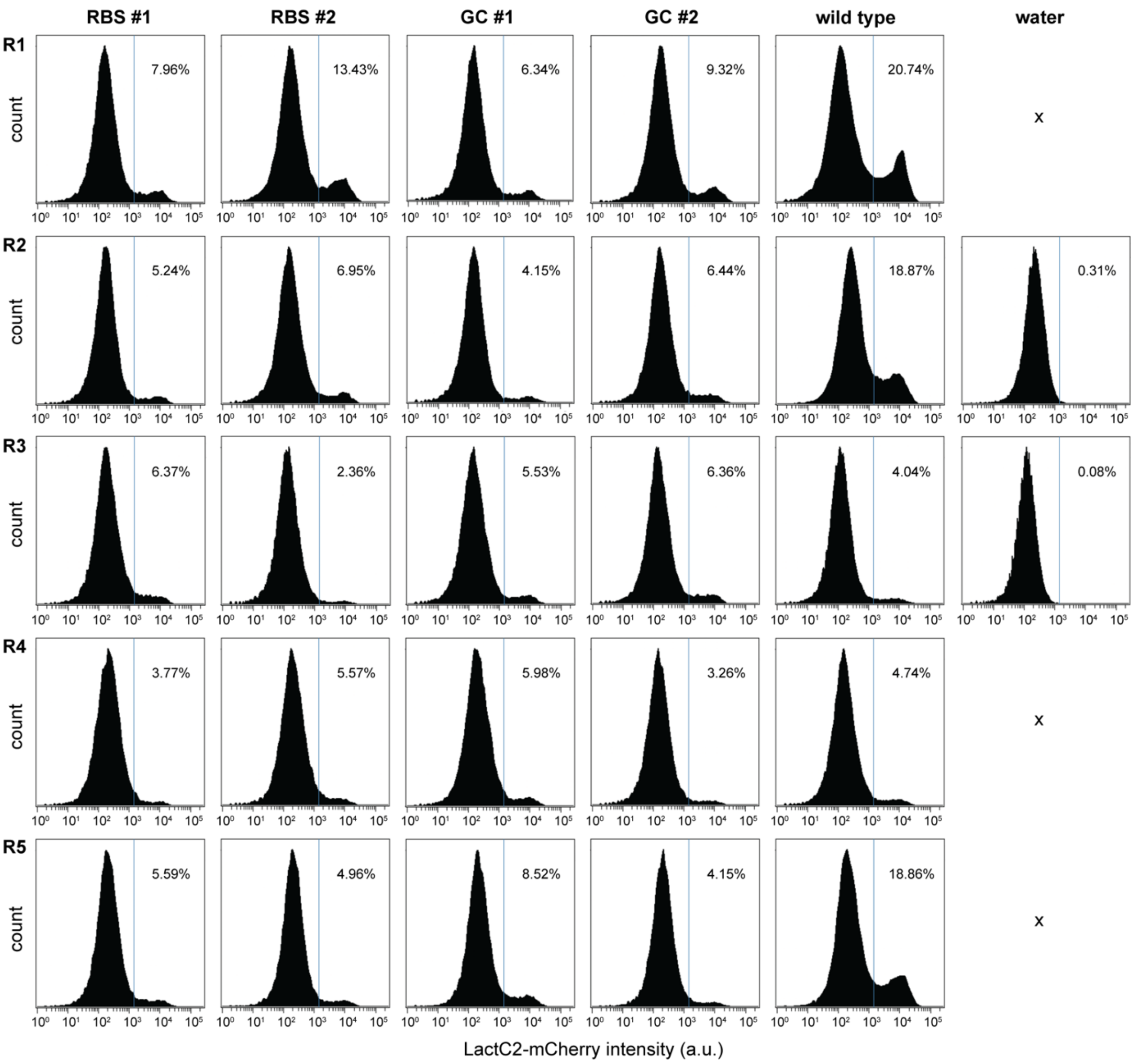

Fig. S10 Histograms displaying the distribution of LactC2-mCherry intensity of total liposome population. Liposomes exhibiting an intensity above the threshold, indicated with blue vertical line, are considered PS-producing liposomes. For the RBS and GC libraries, two biological replicates were analyzed, indicated by #1 and #2.

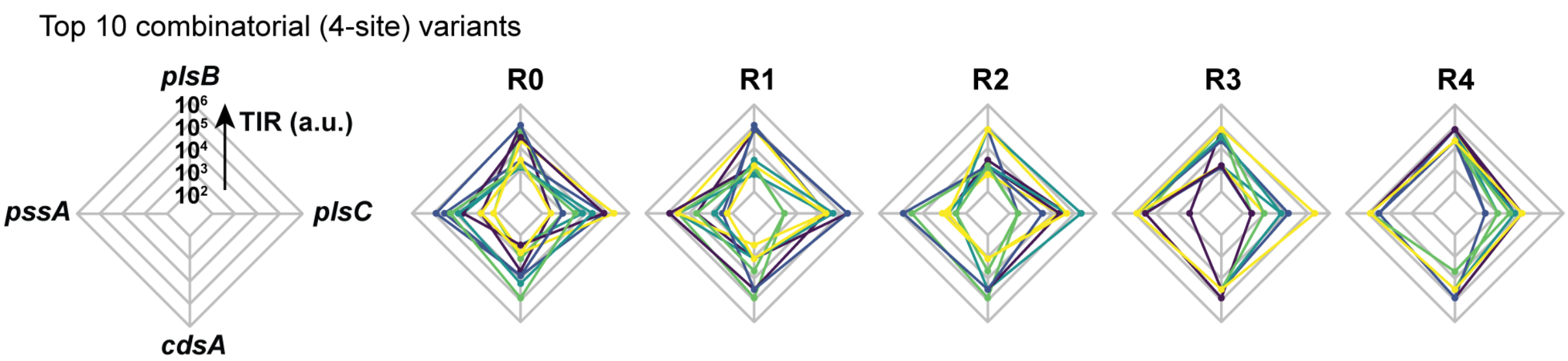

Fig. S11 Radar charts displaying the top 10 most frequent DNA variants for the phospholipid pathway RBS library for sorting round zero to four. The top 10 was derived from the sequencing reads containing a library variant for each RBS (i.e., 4-site library fraction).

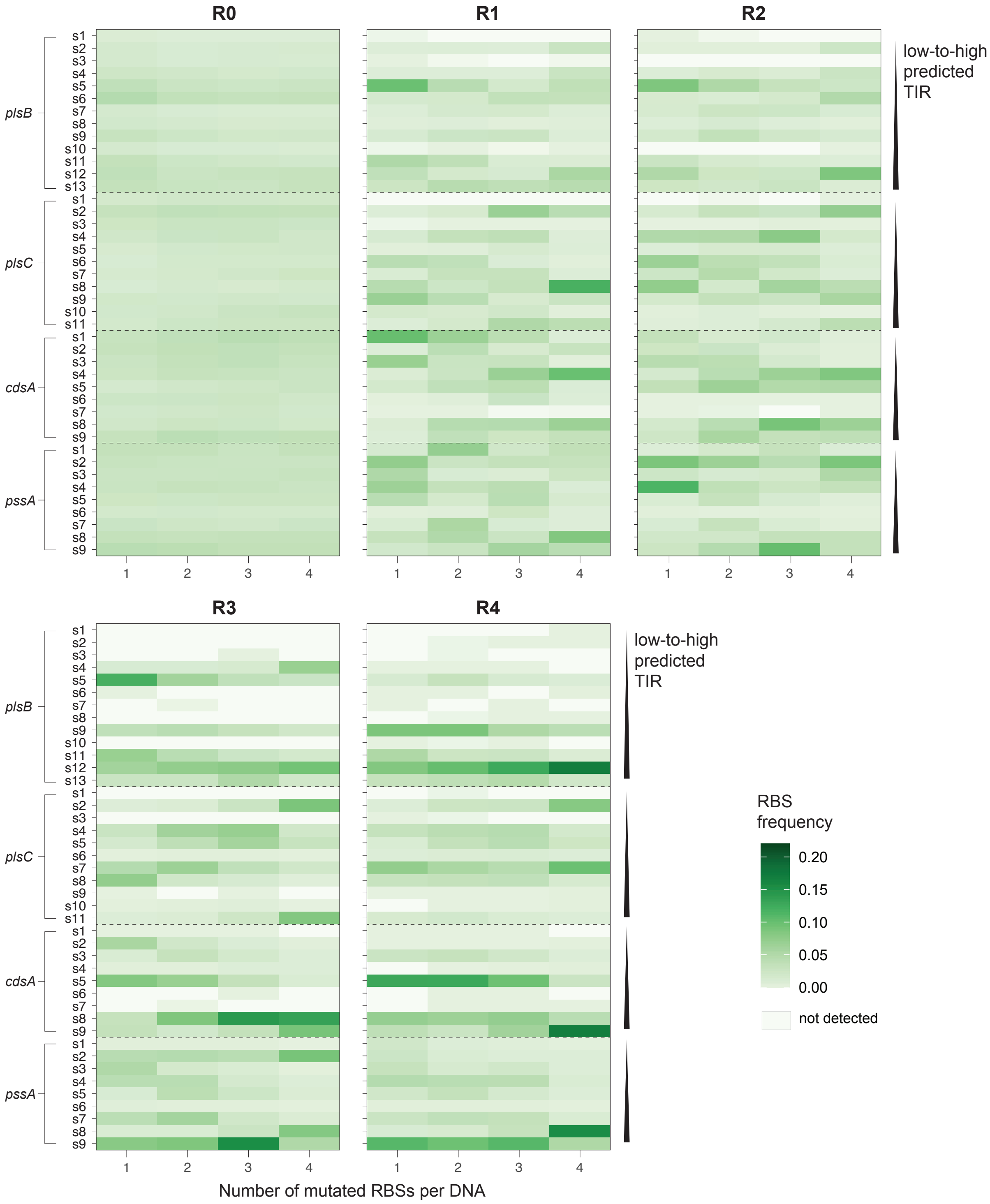

Fig. S12 Heatmaps showing the frequency of each variant of the PS synthesis RBS library for each round. The frequency was calculated relative to the library fractions with n number of mutated RBSs per DNA. The heatmap for R4 is also shown in **Fig. 2c**. Number of reads used to calculate the frequency are noted in **Table S12**.

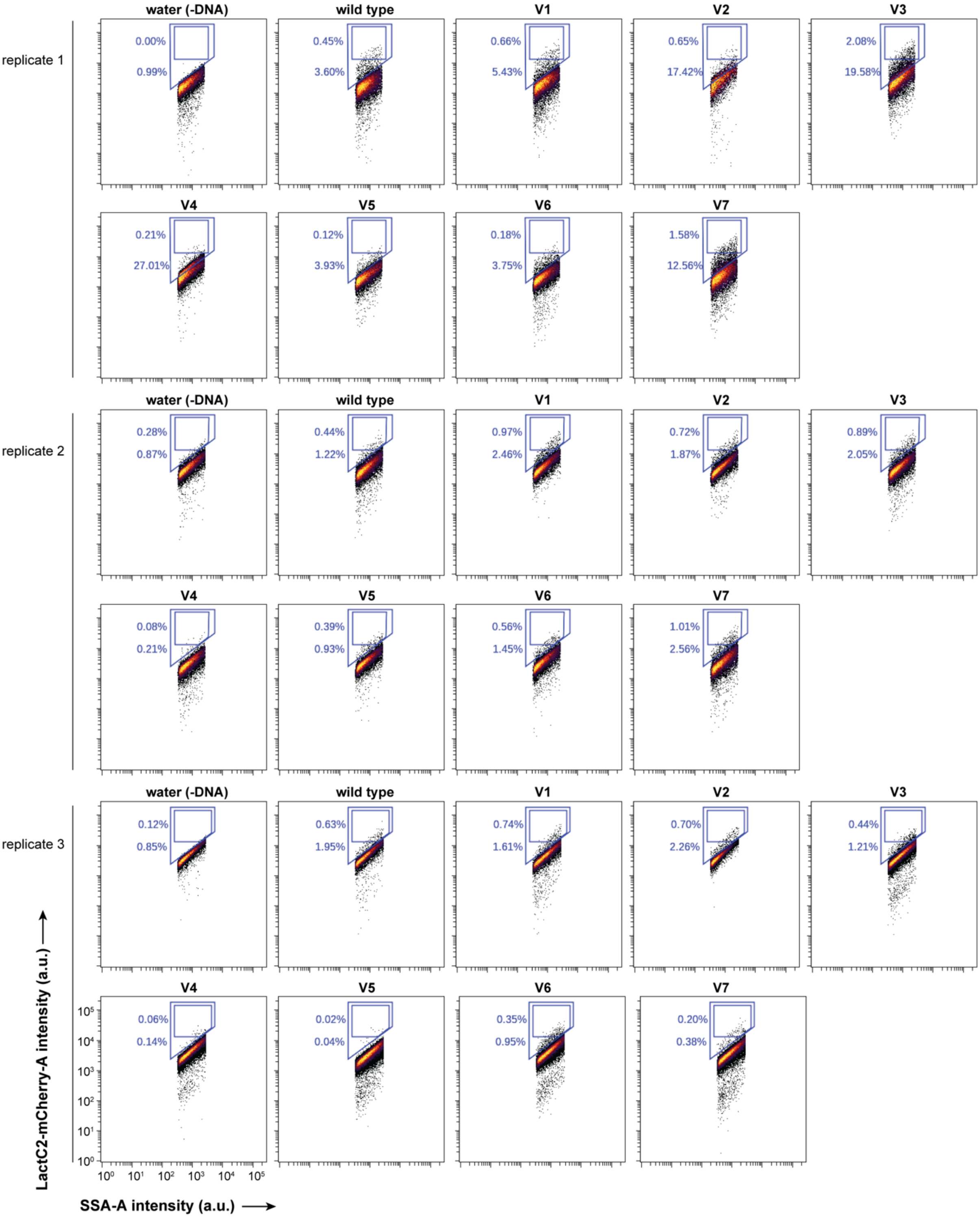

Fig. S13 Flow cytometry measurements of clonal DNA variants V1-7 for phospholipid synthesis. 20,000 events per sample (except plot for V2 of replicate 1 which shows 10,000 events).

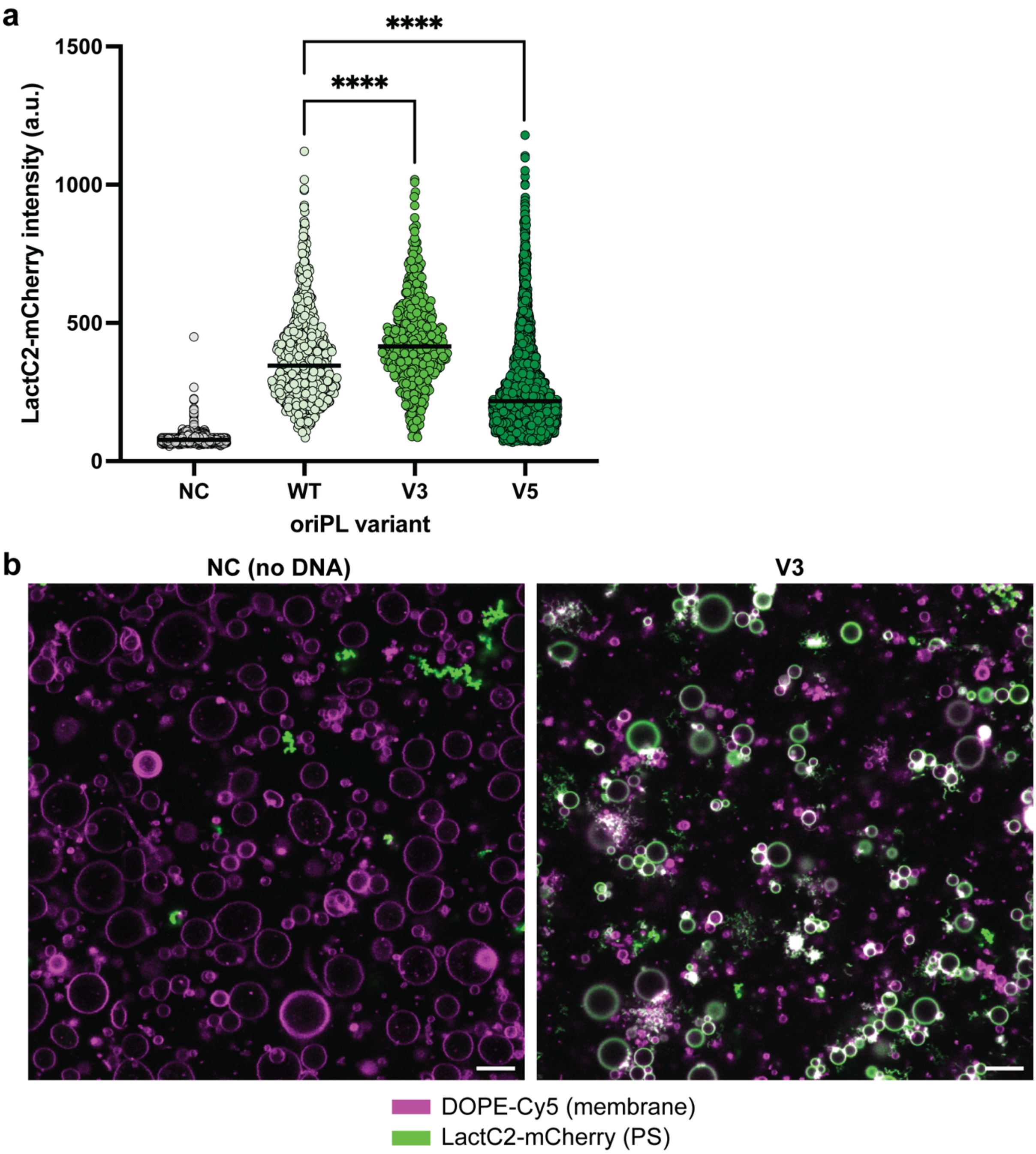

Fig. S14 Confocal image analysis of LactC2-mCherry fluorescence intensity with different DNA variants. 1 nM linear DNA was encapsulated in liposomes together with PURE system. The liposomes were incubated for 12 hours at 37 °C. The negative control (NC) contained MilliQ water instead of DNA. **a** Fluorescence intensity profiles from individual liposomes for different samples. Horizontal line indicates the median. Unpaired *t*-test: **** $P < 0.0001$. Analyzed liposomes per sample: 4,188 (NC), 859 (WT), 476 (V3) and 3,566 (V5). **b** Representative confocal fluorescence microscopy images for the negative control (NC) and V3. Scale bars: 10 µm.

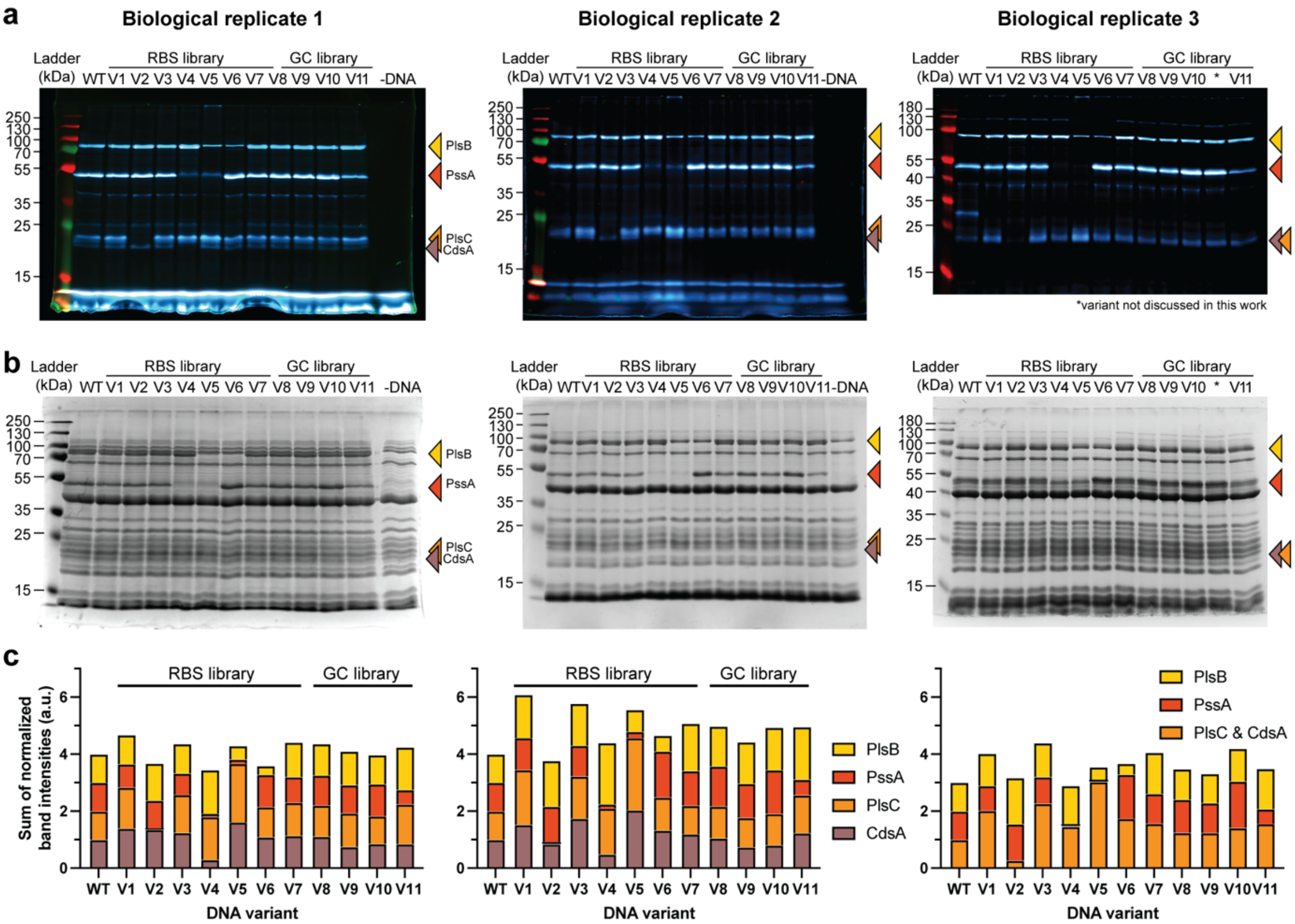

Fig. S15 Bulk PURE reactions with GreenLys protein labeling for the four phospholipid synthesis enzymes with different RBSs (clonal variants V1-7) or GC content at the start of the coding region (clonal variants V8-11). The variant sequences and predicted TIRs are listed in Tables S5 and S9. **a** Fluorescence imaging of SDS-PAGE gels for visualization of the bands corresponding to the proteins expressed by PURE system. The gel for replicate 1 is also displayed in Fig. 2e. **b** Coomassie staining of the same gels shown in panel a to visualize the bands of all proteins. **c** Quantification of the band intensities relative to the wild type (WT). In the third replicate, the amounts of PlsC and CdsA were measured at once as the bands could not be distinguished from each other. Note that V9 and V11 have 4 (unintended) deletions upstream the *pssA* gene, and 1 deletion upstream the *plsB* gene, respectively, which may bias the interpretation of the effect of the targeted GC mutations for these variants.

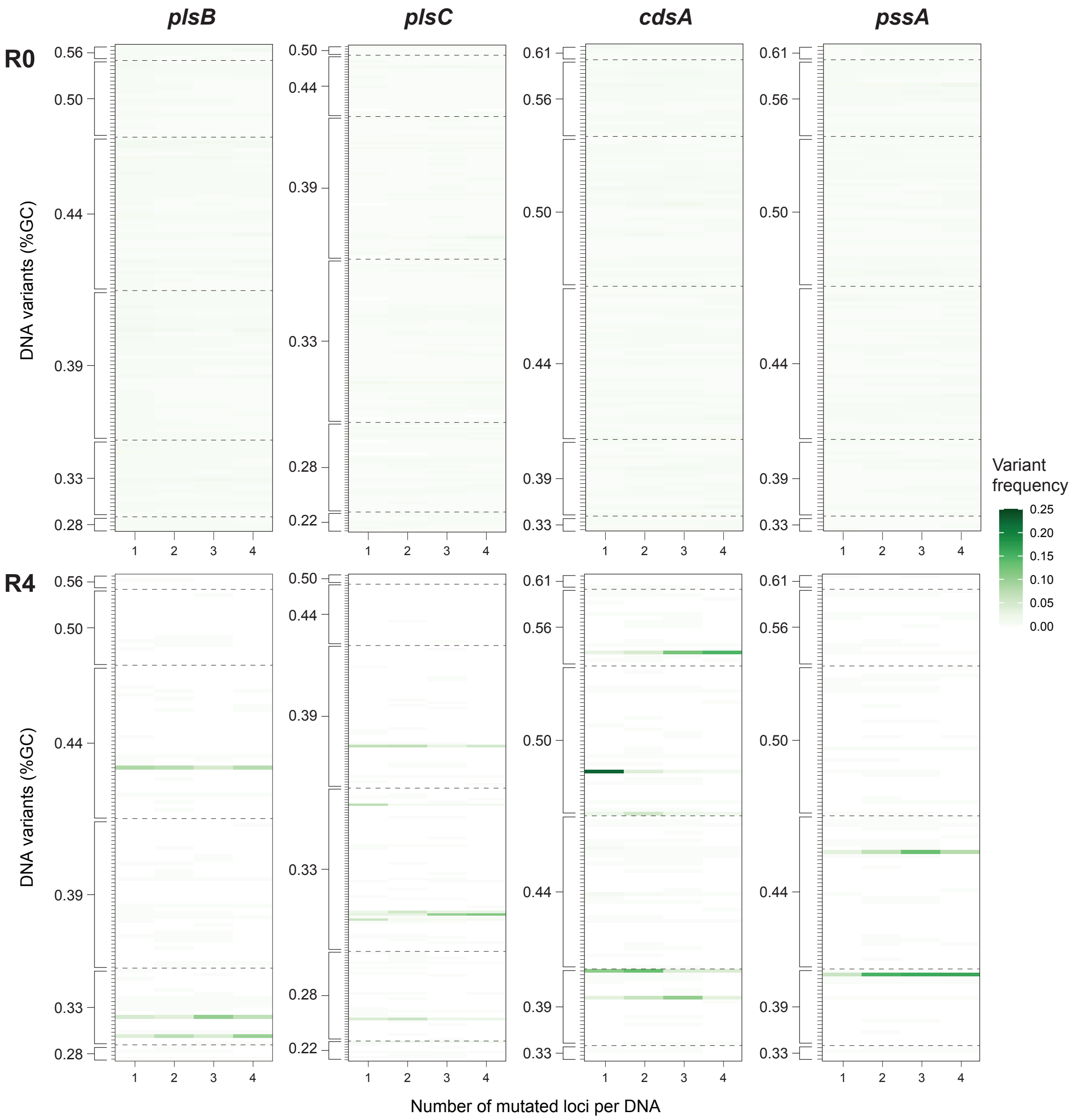

Fig. S16 Heatmaps showing the frequency of each DNA variant in the PS synthesis GC library for R0 (top row) and R4 (bottom row). The frequency was calculated relative to the library fractions with n number of mutated RBSs per DNA. Each tick mark on the y-axis corresponds to one DNA variant (127 DNA variants for *plsB*, *cdsA* and *pssA*, and 191 variants for *plsC*). The DNA variants are grouped based on the GC content of the first six codons of the genes, which ranged from 28-56% for *plsB*, 22-50% for *plsC* and 33-61% for *cdsA* and *pssA*. The GC content in the first six codons of wild-type *plsB*, *plsC*, *cdsA*, *pssA* are 39%, 28%, 50% and 50%, respectively. The number of reads used to calculate the frequency are noted in **Table S13**.

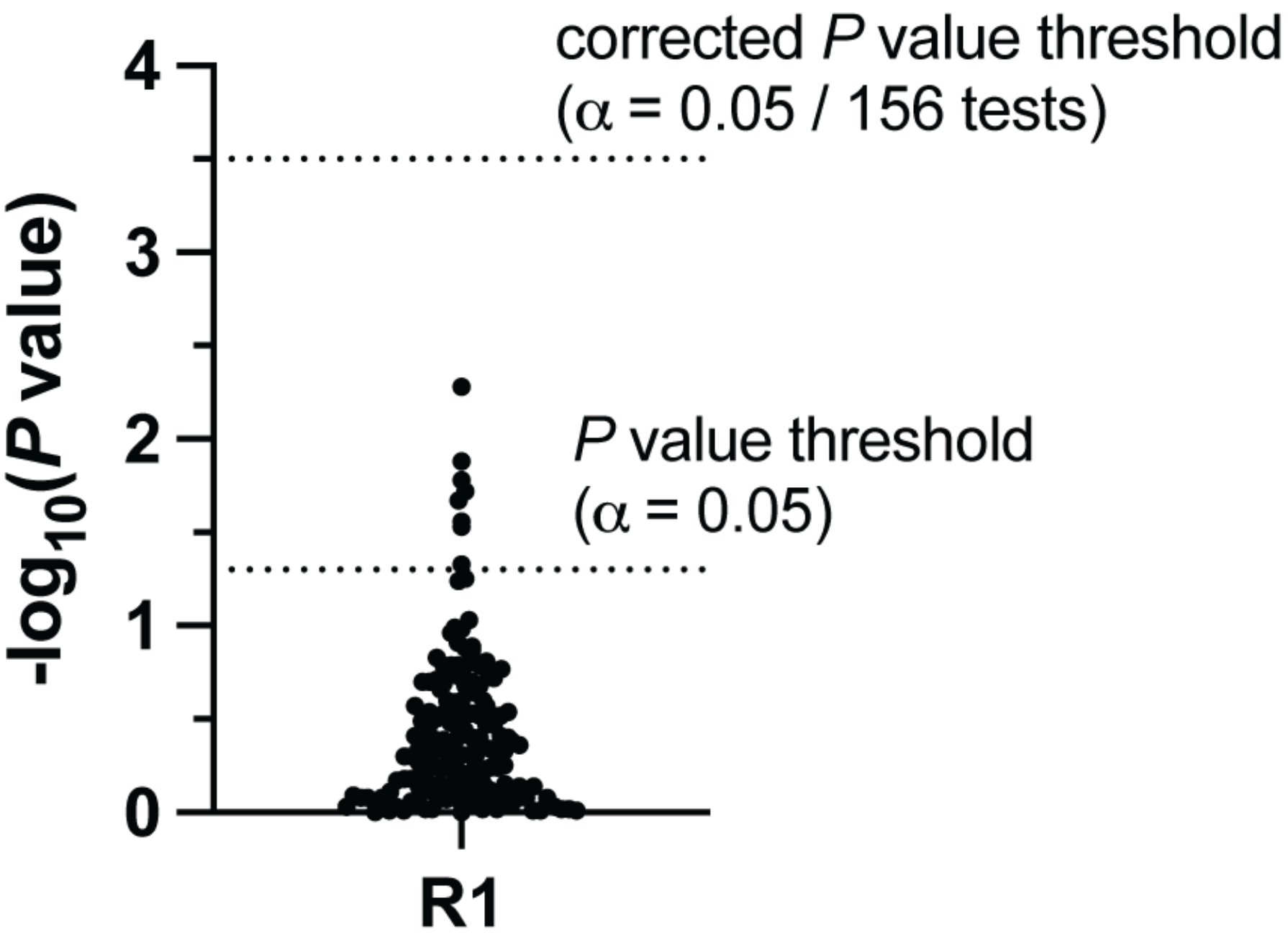

Fig. S17 Statistical analysis of epistatic deviations among the 156 combinatorial DNA replicator variants. Multiple unpaired, two-tailed *t*-tests with null hypothesis: FC_observed – FC_expected = 0 (assuming two-sample unequal variance, n=3 biological replicates). None of the variants exerted significant epistasis (*P* value < 0.05 / 156 tests, Bonferroni correction of the *P* value threshold for multiple comparisons). Exact *P* values, confidence intervals, standard deviation, variance and signal-to-noise ratios of the fold change and/or epistatic deviation are available in the supplemental file 'Data S1'.

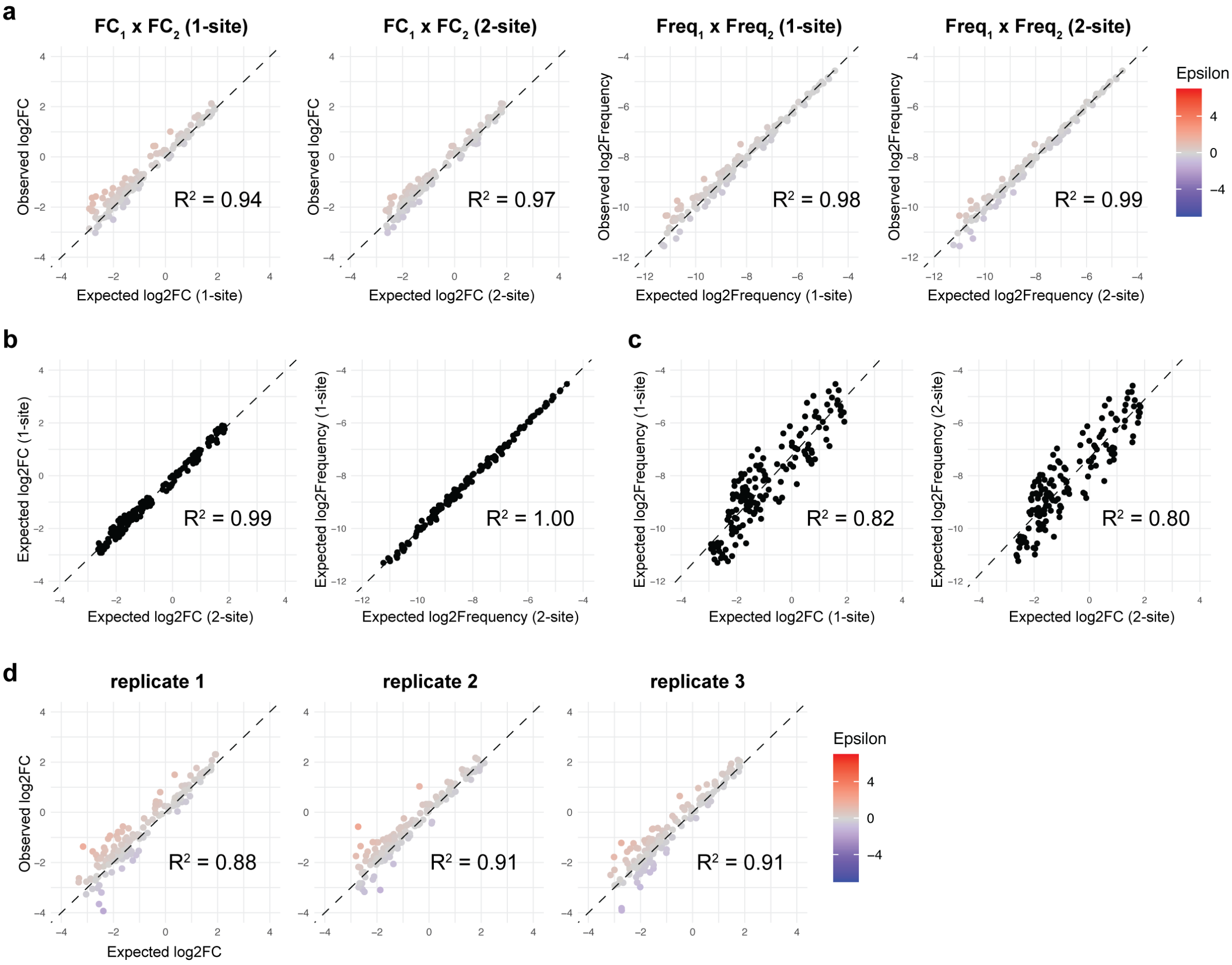

Fig. S18 Different methods for calculating the expected fitness of combinatorial DNA variants for the DNA replicator RBS library. Data of selection round 1 (R1). Dashed line is y = x (panels **a** and **d**) or the result of linear regression (panels **b-c**). All plots contain 156 data points. **a** Observed versus expected fitness plots, where fitness is log2FC or log2Frequency, and the expected fitness was calculated using the 1-site or 2-site library fraction data. Epsilon is the log2 observed fitness – log2 expected fitness. **b** The use of the 1-site or 2-site library fraction to calculate the expected fitness has similar outcomes. **c** Correlation between frequency and fold change for calculating the expected variant fitness. **a-c** Average of three biological replicates. **d** Observed versus expected log2FC of the three individual biological replicates. The expected log2FC was calculated using the 1-site library fraction data.

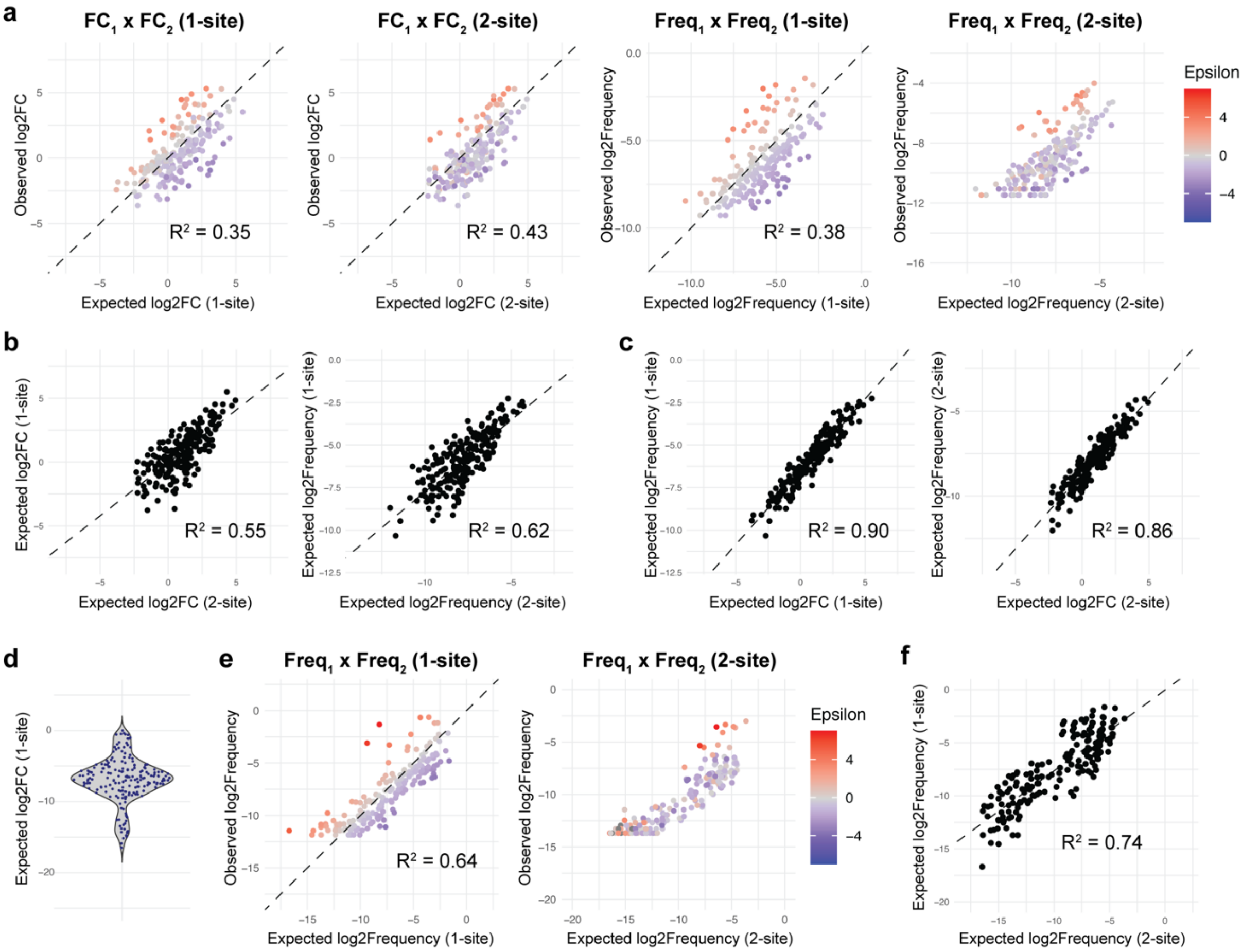

Fig. S19 Different methods for calculating the expected fitness of pairwise combinatorial DNA variants for the PS synthesis RBS and GC libraries. Data of sorting round 4 (R4). Dashed line is y = x (panels **a**, **e**) or the result of linear regression (panels **b-c**, **f**). **a-d** RBS library. **e-f** GC library. **a** Observed versus expected fitness plots for the RBS library, where fitness is log2FC or log2Frequency, and the expected fitness was calculated using the 1-site or 2-site library fraction data. Note that expected log2Frequency (2-site) does not scale with observed log2Frequency in the rightmost plot, because the full combinatorial library (with 4 mutagenic sites) is larger than the pairwise variants. The plotted Epsilon is identical for all plots to show that the high-epistasis variants (in red) are detected by all calculation methods (Epsilon is the observed log2Frequency – expected log2Frequency (1-site) for all plots). **b** Correlation between the expected fitness calculated using the 1-site versus 2-site fraction of the RBS library. **c** The use of FC or Frequency as a measure of the variant fitness leads to similar results. **a-c** All plots show 205 of the 656 pairwise combinatorial variants (DNA variants with less than three reads in the 2-site library fraction were excluded from the analysis). **d** Expected log2FC (calculated using the 1-site library fraction) for combinatorial DNA variants with less than three reads in the 2-site library fraction. The expected log2 fold change was low (mostly negative enrichment factors) compared to the sequenced combinatorial variants in panel a (left plot), explaining why the combinatorial DNA variants were not sequenced. 200 variants were plotted. **e** Observed versus expected fitness plots for the GC library. Note that expected log2Frequency (2-site) does not scale with observed log2Frequency in the right plot, because the full combinatorial library (with 4 mutagenic sites) is larger than the pairwise variants. The plotted Epsilon is identical for the two plots to show that the high-epistasis variants (in red) are detected by the two calculation methods (Epsilon is the observed log2Frequency –expected log2Frequency (1-site)). The left plot is identical to Fig. 4e. **f** Correlation between the expected fitness calculated using the 1-site versus 2-site fraction of the GC library. **e-f** 189 of the 121,158 pairwise combinatorial variants are plotted.

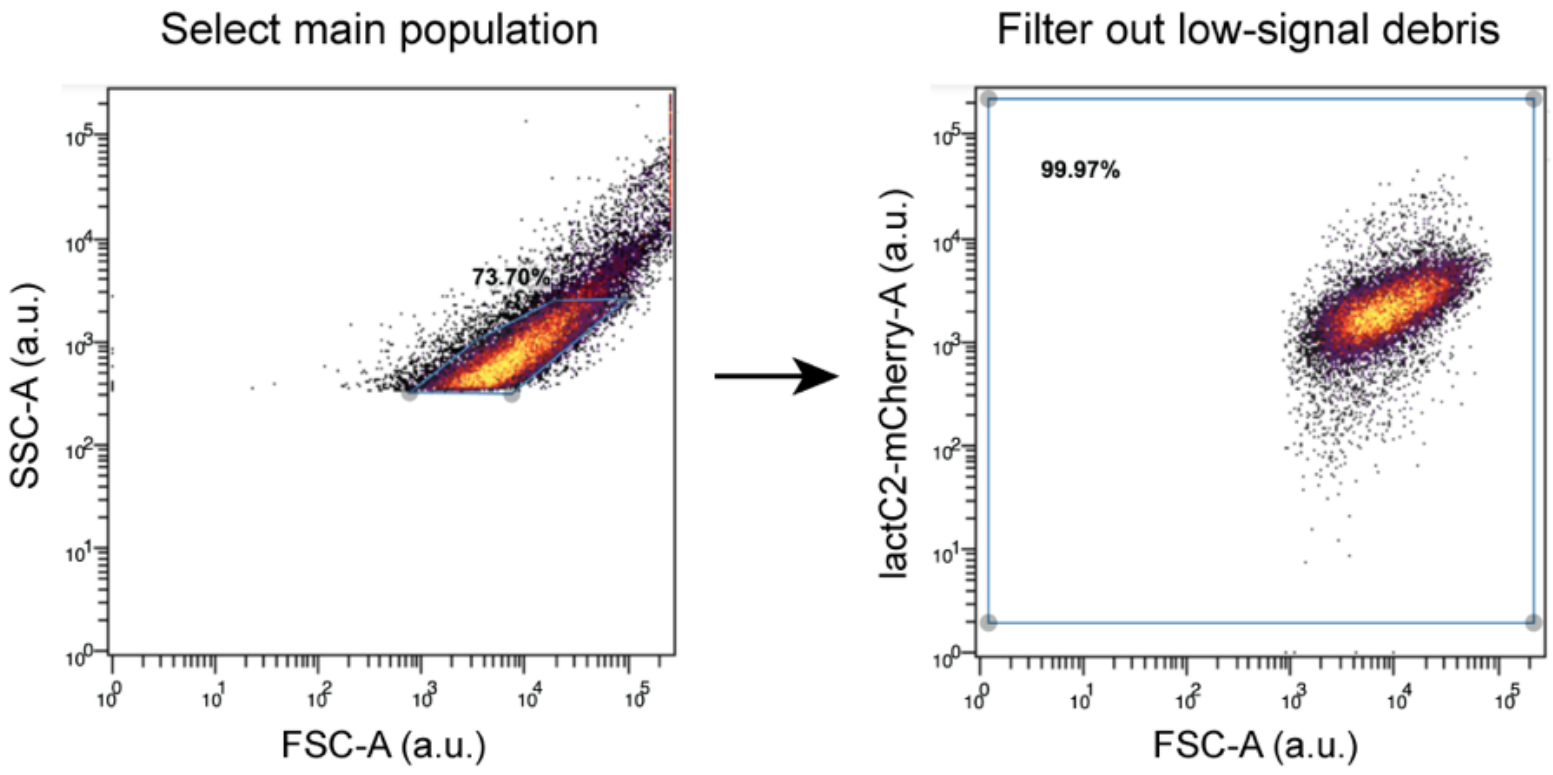

Fig. S20 Example of gating strategy for flow cytometry data. Only the first step (gating based on forward and side scatter light) was applied during sorting. The two filtering steps were applied during post-measurement analysis.

Table S1 List of RBS sequences for DNAP and TP in orip2p3 DNA template. Red color indicates degenerate nucleotides.

	DNAP RBS		TP RBS	
	**Sequence**	**Predicted TIR**	**Sequence**	**Predicted TIR**
Wild type	TTTAAGAAGGAGATATACAT	9,123	TTTAAGAAGGAGATATACTA	25,228
Library degenerate sequence	TTTAADAMGGAGSTATACAT	-	TTTAAGAAGSRGBTTGACTA	-
s1	TTTAAAACGGAGCTATACAT	362	TTTAAGAAGCGGCTTGACTA	110
s2	TTTAAGACGGAGCTATACAT	543	TTTAAGAAGCGGTTTGACTA	146
s3	TTTAATACGGAGCTATACAT	555	TTTAAGAAGCGGGTTGACTA	477
s4	TTTAAAAAGGAGCTATACAT	3,329	TTTAAGAAGCAGTTTGACTA	801
s5	TTTAAGAAGGAGCTATACAT	4,992	TTTAAGAAGCAGCTTGACTA	801
s6	TTTAATAAGGAGCTATACAT	6,933	TTTAAGAAGCAGGTTGACTA	1,476
s7	TTTAAGACGGAGGTATACAT	16,525	TTTAAGAAGGGGCTTGACTA	3,425
s8	TTTAAAACGGAGGTATACAT	16,525	TTTAAGAAGGAGCTTGACTA	4,909
s9	TTTAATACGGAGGTATACAT	17,920	TTTAAGAAGGGGTTTGACTA	8,224
s10	TTTAAGAAGGAGGTATACAT	57,485	TTTAAGAAGGAGTTTGACTA	9,818
s11	TTTAAAAAGGAGGTATACAT	57,485	TTTAAGAAGGGGGTTGACTA	26,545
s12	TTTAATAAGGAGGTATACAT	86,579	TTTAAGAAGGAGGTTGACTA	54,294
s13	-	-	TTTAAGAAGGAGGTTTACTA	110,313

Table S2 Clonal DNA variants from orip2p3 RBS library. The *plsC* RBS of V3 (with predicted TIR: 938 a.u.) was not a variant from the theoretical library, but likely originated from a mutation during MOSAIC or PCR.

	DNAP RBS		TP RBS	
	**Sequence**	**Predicted TIR**	**Sequence**	**Predicted TIR**
WT	WT	9,123	WT	25,228
V1	s10	57,485	s13	110,313
V2	s12	86,579	s12	54,294
V3	TTTAATAAGGAGATATACAT	14,117	s13	110,313
V4	s3	555	s3	477
V5	s5	4,992	s4	801

Table S3 List of RBS sequences for *plsB*, *plsC*, *cdsA* and *pssA* in oriPL DNA template. Red color indicates degenerate nucleotides.

	***plsB* RBS**		***plsC* RBS**	
	**Sequence**	**Predicted TIR**	**Sequence**	**Predicted TIR**
Wild type	TTTAAGAAGGAGATATACAT	5,896	TTTAAGAAGGAGATATACAT	4,947
Library degenerate sequence	TTTAAGGAGGKAAAMBCCAT	-	TTTAAGGVGAATADATACAT	-
s1	TTTAAGGAGGGAAACCCCAT	246	TTTAAGGCGAATAGATACAT	144
s2	TTTAAGGAGGGAAACTCCAT	608	TTTAAGGCGAATATATACAT	249
s3	TTTAAGGAGGGAAACGCCAT	981	TTTAAGGCGAATAAATACAT	878
s4	TTTAAGGAGGGAAAACCCAT	1,309	TTTAAGGGGAATAGATACAT	3,327
s5	TTTAAGGAGGGAAAAGCCAT	1,561	TTTAAGGGGAATATATACAT	5,725
s6	TTTAAGGAGGGAAAATCCAT	2,803	TTTAAGGAGAATAGATACAT	7,182
s7	TTTAAGGAGGTAAACCCCAT	6,346	TTTAAGGAGAATATATACAT	12,360
s8	TTTAAGGAGGTAAACTCCAT	15,661	TTTAAGGGGAATAAATACAT	20,220
s9	TTTAAGGAGGTAAAACCCAT	21,409	TTTAAGGAGAATAAATACAT	43,653
s10	TTTAAGGAGGTAAACGCCAT	25,289	TTTAAGGAGGTAAGATACAT	68,987
s11	TTTAAGGAGGTAAAAGCCAT	31,839	TTTAAGGAGGTAATATACAT	201,909
s12	TTTAAGGAGGTAAAATCCAT	72,247		
s13	TTTAAGGAGGTAATATACAT	112,986		

	***cdsA* and *pssA* RBSs**	
	**Sequence**	**Predicted TIR**
Wild type	TTTAAGAAGGAGATATACAT	5,198
Library degenerate sequence	TTTAAGARKGAGKTATACAT	-
s1	TTTAAGAATGAGTTATACAT	174
s2	TTTAAGAGTGAGTTATACAT	289
s3	TTTAAGAGGGAGTTATACAT	659
s4	TTTAAGAAGGAGTTATACAT	1,231
s5	TTTAAGAATGAGGTATACAT	4,501
s6	TTTAAGAGTGAGGTATACAT	7,484
s7	TTTAAGAGGGAGGTATACAT	17,054
s8	TTTAAGAAGGAGGTATACAT	31,879
s9	GATAAGGAGGTGATATACAT	78,824

Table S4 Calculation of FACS screening coverage for RBS library. Calculation based on 4,500,000 screened events and 13% PS-producing liposomes (for wild type). Calculation example (row 2): Per-variant screening coverage = 4,500,000 (number of events) * 0.23 (frequency of this library fraction of total library) * 0.13 (% PS-producing liposomes) / 42 (number of variants) = 3,204.

**Library fraction**	**Number of mutated RBSs in this library fraction**	**Number of variants**	**Fraction of total library**	**Per-variant screening coverage**
1	0 (WT)	1	0.12	70,200
2	1	42	0.23	3,204
3	2	656	0.29	259
4	3	4,518	0.25	32
5	4	11,583	0.11	6

Table S5 Clonal DNA variants from oriPL RBS library.

	plsB RBS		*plsC* RBS		*cdsA* RBS		*pssA* RBS	
	**Sequence**	**Predicted TIR**	**Sequence**	**Predicted TIR**	**Sequence**	**Predicted TIR**	**Sequence**	**Predicted TIR**
WT	WT	5,896	WT	4,947	WT	5,198	WT	5,198
V1	s12	72,247	s7	12,360	s9	78,824	s9	78,824
V2	s12	72,247	TTTAAGG<br>GGAATAG<br>ATATAT	938	s9	78,824	s9	78,824
V3	s12	72,247	s11	201,909	s9	78,824	s9	78,824
V4	WT	5,896	WT	4,947	s1	174	s1	174
V5	s4	1,309	s7	12,360	WT	5,198	s1	174
V6	s4	1,309	WT	4,947	WT	5,198	WT	5,198
V7	WT	5,896	WT	4,947	WT	5,198	s9	78,824

Table S6 Top 10 enriched variants in the 4-site fraction of the RBS oriPL library after round 4.

***plsB* RBS sequence**	***plsC* RBS sequence**	***cdsA* RBS sequence**	***pssA* RBS sequence**	***plsB* RBS TIR**	***plsC* RBS TIR**	***cdsA* RBS TIR**	***pssA* RBS TIR**	**Number of reads**
TTTAAGGAGGT AAAATCCAT	TTTAAGGAGAA TATATACAT	GATAAGGAGGT GATATACAT	TTTAAGAAGGA GGTATACAT	72,247	12,360	78,824	31,879	181
TTTAAGGAGGT AAAATCCAT	TTTAAGGCGAA TATATACAT	GATAAGGAGGT GATATACAT	TTTAAGAAGGA GGTATACAT	72,247	249	78,824	31,879	172
TTTAAGGAGGT AAAATCCAT	TTTAAGGGGAA TAGATATAT	TTTAAGAAGGA GGTATACAT	GATAAGGAGGT GATATACAT	72,247	938	31,879	78,824	162
TTTAAGGAGGT AAAACCCAT	TTTAAGGGGAA TAGATATAT	TTTAAGAAGGA GGTATACAT	GATAAGGAGGT GATATACAT	21,409	938	31,879	78,824	18
TTTAAGGAGGT AAAATCCAT	TTTAAGGGGAA TAGATACAT	TTTGAGAAGGA GATATACAT	GATAAGGAGGT GATATACAT	72,247	3,327	4,794	78,824	17
TTTAAGGAGGT AAAATCCAT	TTTAAGGGGAA TATATACAT	TTTAAGAAGGA GGTATACAT	GATAAGGAGGT GATATACAT	72,247	5,725	31,879	78,824	16
TTTAAGGAGGT AAAACCCAT	TTTAAGGCGAA TATATACAT	GATAAGGAGGT GATATACAT	TTTAAGAAGGA GGTATACAT	21,409	249	78,824	31,879	15
TTTAAGGAGGT AAAACCCAT	TTTAAGGGGAA TATATACAT	TTTAAGAAGGA GGTATACAT	GATAAGGAGGT GATATACAT	21,409	5,725	31,879	78,824	14
TTTAAGGAGGT AAAACCCAT	TTTAAGGGGAA TAGATACAT	TTTGAGAAGGA GATATACAT	GATAAGGAGGT GATATACAT	21,409	3,327	4,794	78,824	13
TTTAAGGAGGT AAAACCCAT	TTTAAGGAGAA TATATACAT	TTTAAGAAGGA GGTATACAT	GATAAGGAGGT GATATACAT	21,409	12,360	31,879	78,824	11

Table S7 Calculation of FACS screening coverage for the theoretical GC library. Calculation based on 4,500,000 screened events and 13% PS-producing liposomes (for wild type). With a MOSAIC editing efficiency of ~50% per target locus, we obtained a library that consisted for 9% of variants with all target loci mutated, and the remainder of the library consisted of library fractions where zero, one, two or three loci were mutated (**Fig. S11a**). Using nanopore sequencing, we detected 565, 9,236, 7,785 and 2,922 library variants with one, two, three and four mutated RBSs, respectively, in the initial library. This corresponded to 99%, 8%, 0.1% and 0.001% of all theoretical library variants with one, two, three and four mutated RBSs, respectively. Potentially, more of the theoretical library is covered but not detected with the current sequencing depth.

**Screening coverage for DNA variants:**				
**Library fraction**	**Number of mutated loci**	**Number of DNA variants**	**Fraction of total library**	**Per-variant screening coverage**
1	0 (WT)	1	0.14	81,900
2	1	572	0.25	256
3	2	121,158	0.29	1
4	3	11,290,300	0.23	0.01
5	4	391,241,153	0.09	0.0001
**Screening coverage for %GC variants:**				
**Library fraction**	**Number of mutated loci**	**Number of %GC variants**	**Fraction of total library**	**Per-variant screening coverage**
1	0 (WT)	1	0.14	81,900
2	1	6	0.25	24,375
3	2	36	0.29	4,713
4	3	216	0.23	623
5	4	1,296	0.09	41

Table S8 Most enriched variants (variant frequency > 0.02) of the GC oriPL library after round 4.

**Gene**	**DNA sequence**	**Predicted TIR (a.u.)**
plsB	CTTTTGTTATCCA	5,870
plsB	ATTTTGCTATCCA	20,695
plsB	CTTTTGTTACCCC	5,870
plsC	ATATATCTTTCGC	8,605
plsC	TTATATATTCCGG	34,414
plsC	CTACATTTTCCGA	21,844
plsC	ATATATCTTCCGA	2,975
plsC	ATATATCTTTCGA	4,859
plsC	ATATATCTTTCGG	6,809
cdsA	CAGCATTACCGT	13,195
cdsA	CAGCATGACCGT	10,073
cdsA	TAGTATTACTGG	13,618
cdsA	CAGCATTACCGT	13,195
cdsA	CAGCATGACCGT	10,073
pssA	TAGTATTACGGT	5,611
pssA	TAGCATTACAGG	14,700

Table S9 Clonal DNA variants from the oriPL GC library.

	***plsB* RBS**			***plsC* RBS**		
	**Sequence**	**%GC**	**Predicted TIR**	**Sequence**	**%GC**	**Predicted TIR**
WT	WT (TTTCTGCTATCCT)	0.38	5,896	WT (ATATATCTTTCGT)	0.23	4,947
V8	ATTTTGCTATCCA	0.31	20,695	ATATATCTTTCGC	0.31	8,605
V9	ATTTTGCTATCCA	0.31	20,695	ATATATCTTTCGC	0.31	8,605
V10	ATTTTGCTATCCA	0.31	20,695	ATATATCTTTCGC	0.31	8,605
V11$	ATTTTGCTATCCA	0.31	12,389$	ATATATCTTTCGC	0.31	8,605

	***cdsA* RBS**			***pssA* RBS**		
	**Sequence**	**%GC**	**Predicted TIR**	**Sequence**	**%GC**	**Predicted TIR**
WT	WT (TAGCATGACTGG)	0.50	5,198	WT (TAGCATGACTGG)	0.50	5,198
V8	WT	0.50	5,198	WT	0.50	5,198
V9	CAGCATGACCGT	0.58	10,073	WT*	0.50	9,289*
V10	TAGTATTACTGT	0.25	13,618	TAGTATTACTGT	0.25	13,618
V11	CAGCATGACCGT	0.58	10,073	CAGCATGACCGT	0.58	10,073

$ V11 has a deletion at the 22nd nucleotide upstream the start codon of the *plsB* gene.
* V9 has 4 deletions 17-20 nucleotides upstream the start codon of the *pssA* gene.

Table S10 List of plasmids.

Plasmid name	Description
G555	Expression of PlsB, PlsC, CdsA and PssA under the control of T7 promoters. The 4-gene insert is flanked by Phi29 replication origins. Addgene #216483.
G435	Expression of DNAP and TP under the control of T7 promoters. The 2-gene insert is flanked by Phi29 replication origins.
pORTMAGE-Ec1	Enables high-efficiency plasmid and genomic recombineering in *E. coli*. Expresses single-stranded DNA annealing protein (SSAP) CspRecT and the dominant negative *E. coli* MutL mutant (EcMutL^{E32K}), controlled by XylS-Pm expression system. Addgene #138474.

Table S11 List of DNA oligos. '*' indicates phosphorothioate bond. Red color indicates degenerate nucleotides.

Primer name	Sequence (5′ to 3′)	Description
DNAP DEG	C*A*CAACGGTTTCCCTCTAGAAA TAATTTTGTTTAACTTTAADAMG GAGSTATACATATGCCGCGTAAA ATGTACAGCTGCGATTTTGAAAC	G435 RBS library<br>Degenerate MOSAIC oligo for mutating the *DNAP* RBS in plasmid G435.
TP DEG	G*A*CACCAGAGGGTTTACATGTT TATTTGTTTAACTTTAAGAAGSRG BTTGACTAATGGCACGCAGCCCG CGCATCCGCATCAAAGATAACG	G435 RBS library<br>Degenerate MOSAIC oligo for mutating the *TP* RBS in plasmid G435.

Table S11 continued

TP_1 custom	G*A*CACCAGAGGGTTTACATGTT TATTTGTTTAACTTTAAGAAGGAG GTTTACTAATGGCACGCAGCCCG CGCATCCGCATCAAAGATAACG	G435 RBS library<br>Additional MOSAIC oligo for mutating the *TP* RBS in plasmid G435. This oligo was mixed with the degenerate *TP* oligo set before addition to the electroporation sample.
1585 ChD	C*A*CAACGGTTTCCCTCTAGAAA TAATTTTGTTTAACTTTAAGAAGG AGGTATACATATGCCGCGTAAAA TGTACAGCTGCGATTTTGAAAC	G435 RBS variant (s10) targeting the *DNAP* RBS (MOSAIC oligo).
1586 ChD	C*A*CAACGGTTTCCCTCTAGAAA TAATTTTGTTTAACTTTAATAAGG AGGTATACATATGCCGCGTAAAA TGTACAGCTGCGATTTTGAAAC	G435 RBS variant (s12) targeting the *DNAP* RBS (MOSAIC oligo).
1587 ChD	C*A*CAACGGTTTCCCTCTAGAAA TAATTTTGTTTAACTTTAATACGG AGCTATACATATGCCGCGTAAAA TGTACAGCTGCGATTTTGAAAC	G435 RBS variant (s3) targeting the *DNAP* RBS (MOSAIC oligo).
1588 ChD	C*A*CAACGGTTTCCCTCTAGAAA TAATTTTGTTTAACTTTAAGAAGG AGCTATACATATGCCGCGTAAAA TGTACAGCTGCGATTTTGAAAC	G435 RBS variant (s5) targeting the *DNAP* RBS (MOSAIC oligo).
1589 ChD	C*A*CAACGGTTTCCCTCTAGAAA TAATTTTGTTTAACTTTAATAAGG AGATATACATATGCCGCGTAAAA TGTACAGCTGCGATTTTGAAAC	G435 RBS variant targeting the *DNAP* RBS (MOSAIC oligo).
1590 ChD	G*A*CACCAGAGGGTTTACATGTT TATTTGTTTAACTTTAAGAAGGAG GTTGACTAATGGCACGCAGCCCG CGCATCCGCATCAAAGATAACG	G435 RBS variant (s12) targeting the *TP* RBS (MOSAIC oligo).
1591 ChD	G*A*CACCAGAGGGTTTACATGTT TATTTGTTTAACTTTAAGAAGCGG GTTGACTAATGGCACGCAGCCCG CGCATCCGCATCAAAGATAACG	G435 RBS variant (s3) targeting the *TP* RBS.
1592 ChD	G*A*CACCAGAGGGTTTACATGTT TATTTGTTTAACTTTAAGAAGCAG TTTGACTAATGGCACGCAGCCCG CGCATCCGCATCAAAGATAACG	G435 RBS variant (s4) targeting the *TP* RBS (MOSAIC oligo).
PlsB DEG	C*A*ACGGTTTCCCTCTAGAAATA ATTTTGTTTAACTTTAAGGAGGKA AAMBCCATATGACTTTCTGCTAT CCTTGCCGCGCATTTGCATTA	G555 RBS library<br>Degenerate MOSAIC oligo for mutating the *plsB* RBS in plasmid G555.
PlsC DEG	C*A*ACGGTTTCCCTCTAGAAATA ATTTTGTTTAACTTTAAGGVGAAT ADATACATATGCTATATATCTTTC GTCTTATTATTACCGTGATT	G555 RBS library<br>Degenerate MOSAIC oligo for mutating the *plsC* RBS in plasmid G555.
PssA_CdsA DEG	A*T*AACAATTCCCCTCTAGAAAT AATTTTGTTTAACTTTAAGARKGA GKTATACATATGGCTAGCATGAC TGGTGGACAGCAAATGGGTCG	G555 RBS library<br>Degenerate MOSAIC oligo for mutating the *cdsA and pssA* RBSs in plasmid G555.

Table S11 continued

PlsB custom	C*A*ACGGTTTCCCTCTAGAAATA ATTTTGTTTAACTTTAAGGAGGTA ATATACATATGACTTTCTGCTATC CTTGCCGCGCATTTGCATTA	G555 RBS library<br>Additional MOSAIC oligo for mutating the *plsB* RBS in plasmid G555. This oligo was mixed with the degenerate *plsB* oligo set before addition to the electroporation sample.
PlsC_1 custom	C*A*ACGGTTTCCCTCTAGAAATA ATTTTGTTTAACTTTAAGGAGGTA ATATACATATGCTATATATCTTTC GTCTTATTATTACCGTGATT	G555 RBS library<br>Additional MOSAIC oligo for mutating the *plsC* RBS in plasmid G555. This oligo was mixed with the degenerate *plsC* oligo set before addition to the electroporation sample.
PlsC_2 custom	C*A*ACGGTTTCCCTCTAGAAATA ATTTTGTTTAACTTTAAGGAGGTA AGATACATATGCTATATATCTTTC GTCTTATTATTACCGTGATT	G555 RBS library<br>Additional MOSAIC oligo for mutating the *plsC* RBS in plasmid G555. This oligo was mixed with the degenerate *plsC* oligo set before addition to the electroporation sample.
PssA_CdsA custom	A*G*CGGATAACAATTCCCCTCTA GAAATAATTTTGTTTAACGATAA GGAGGTGATATACATATGGCTAG CATGACTGGTGGACAGCAAATG	G555 RBS library<br>Additional MOSAIC oligo for mutating the *cdsA and pssA* RBSs in plasmid G555. This oligo was mixed with the degenerate *cdsA/pssA* oligo set before addition to the electroporation sample.
1543 ChD	C*A*ACGGTTTCCCTCTAGAAATA ATTTTGTTTAACTTTAAGGAGGTA AAATCCATATGACTTTCTGCTATC CTTGCCGCGCATTTGCATTA	G555 RBS variant (s12) targeting the *plsB* RBS (MOSAIC oligo).
1544 ChD	C*A*ACGGTTTCCCTCTAGAAATA ATTTTGTTTAACTTTAAGGAGGGA AAACCCATATGACTTTCTGCTATC CTTGCCGCGCATTTGCATTA	G555 RBS variant (s4) targeting the *plsB* RBS (MOSAIC oligo).
1545 ChD	C*A*ACGGTTTCCCTCTAGAAATA ATTTTGTTTAACTTTAAGGAGAAT ATATACATATGCTATATATCTTTC GTCTTATTATTACCGTGATT	G555 RBS variant (s7) targeting the *plsC* RBS (MOSAIC oligo).
1547 ChD	C*A*ACGGTTTCCCTCTAGAAATA ATTTTGTTTAACTTTAAGGGGAAT AGATATATATGCTATATATCTTTC GTCTTATTATTACCGTGATT	G555 RBS variant targeting the *plsC* RBS (MOSAIC oligo).
1549 ChD	A*T*AACAATTCCCCTCTAGAAAT AATTTTGTTTAACTTTAAGAATGA GTTATACATATGGCTAGCATGAC TGGTGGACAGCAAATGGGTCG	G555 RBS variant (s1) targeting the *cdsA/pssA* RBSs (MOSAIC oligo).
1481 ChD	A*T*AATTTTGTTTAACTTTAAGAA GGAGATATACATATGACNTTYTG YTAYCCNTGCCGCGCATTTGCAT TATTAACCAGAGGCTTTACAT	G555 GC library<br>Degenerate MOSAIC oligo for modification of the GC content in the first five codons after the start codon of the *plsB* gene in plasmid G555.
1482 ChD	A*T*AATTTTGTTTAACTTTAAGAA GGAGATATACATATGCTNTAYAT HTTYCGNCTTATTATTACCGTGAT TTACAGCATCTTAGTCTGTG	G555 GC library<br>Degenerate MOSAIC oligo for modification of the GC content in the first five codons after the start codon of the *plsC* gene in plasmid G555.

Table S11 continued

1483 ChD	T*C*TAGAAATAATTTTGTTTAACTTTAAGAAGGAGATATACATATGGCNAGYATKACNGKTGGACAGCAAATGGGTCGCGGATCCGGCTGC	G555 GC library<br>Degenerate MOSAIC oligo for modification of the GC content in the first five codons after the start codon of the T7 tag preceding the *cdsA* and *pssA* genes in plasmid G555.
1551 ChD	A*T*AATTTTGTTTAACTTTAAGAAGGAGATATACATATGACATTTTGCTATCCATGCCGCGCATTTGCATTATTAACCAGAGGCTTTACAT	G555 GC variant targeting *plsB* (MOSAIC oligo).
1554 ChD	A*T*AATTTTGTTTAACTTTAAGAAGGAGATATACATATGCTATATATCTTTCGCCTTATTATTACCGTGATTTACAGCATCTTAGTCTGTG	G555 GC variant targeting *plsC* (MOSAIC oligo).
1557 ChD	T*C*TAGAAATAATTTTGTTTAACTTTAAGAAGGAGATATACATATGGCCAGCATGACCGTTGGACAGCAAATGGGTCGCGGATCCGGCTGC	G555 GC variant targeting *cdsA/pssA* (MOSAIC oligo).
1560 ChD	T*C*TAGAAATAATTTTGTTTAACTTTAAGAAGGAGATATACATATGGCTAGTATTACTGTTGGACAGCAAATGGGTCGCGGATCCGGCTGC	G555 GC variant targeting *cdsA/pssA* (MOSAIC oligo).
491 ChD	5’Phos-AAAGTAAGCCCCCACCCTCACATG	PCR primer binding to oriL191 in G435 and G555.
492 ChD	5’Phos-AAAGTAGGGTACAGCGACAACATACAC	PCR primer binding to oriR194 in G435 and G555.
1523 ChD	5’Phos-AAAGTAAGCCCCCACCCTCACATGATACC	Alternative PCR primer for 491 ChD.
1524 ChD	5’Phos-AAAGTAGGGTACAGCGACAACATACACCATTTCC	Alternative PCR primer for 492 ChD.
1459 ChD	CTCCTAATATCGACATAATCCGTCGATCCTCG	PCR primer binding to oriL191 used for recovery of oriPL from liposomes after FACS.
1460 ChD	CCCCATTGACCGACTATCTTCGACAAG	PCR primer binding to oriR194 used for recovery of oriPL from liposomes after FACS.
1301 ChD	5’Phos-AAAGTAAGCCCCCACCCTCACATGATACCATTCTCCTAATATCGACATAATCCGTCGATCC	PCR primer binding to oriL191 used for recovery of oriPL from liposomes after FACS.
1518 ChD	5'Phos-AAAGTAGGGTACAGCGACAACATACACCATTTCCCCATTGACCGACTATCTTCG	PCR primer binding to oriR194 used for recovery of oriPL from liposomes after FACS.
976 ChD	GGATGAAGACTACCCGCTGC	qPCR primer binding in *DNAP*.
977 ChD	ACAGGTCTGCGATTTCACCG	qPCR primer binding in *DNAP*.

Table S12 Total number of reads of the different library fractions (n-site fractions) for the RBS library of the phospholipid synthesis pathway, supplementing the data in **Fig. S12**:

**Round**	**1-site**	**2-site**	**3-site**	**4-site**
**R0**	11,977	15,586	13,835	6,666
**R1**	19,404	17,740	14,456	7,134
**R2**	11,897	13,071	5,189	2,030
**R3**	9,528	7,790	3,268	970
**R4**	9,123	8,510	3,119	697

Table S13 Total number of reads for the different library fractions of the GC library of the phospholipid synthesis pathway, supplementing the data in **Fig. S16**.

**Round**	**1-site**	**2-site**	**3-site**	**4-site**
**R0**	10,314	12,067	9,410	3,607
**R1**	37,127	32,459	17,614	5,850
**R2**	55,764	31,071	14,882	2,988
**R3**	41,912	34,681	12,152	1,309
**R4**	34,025	39,306	17,120	1,639
